# Classical Phytohormones and Peptide Plant Hormones in Abiotic Stress Tolerance: Crosstalk, Physiological Integration, and Crop Improvement

**DOI:** 10.3390/plants15101538

**Published:** 2026-05-18

**Authors:** Baber Ali, Ayesha Imran, Hamza Iftikhar, Zeeshan Khan, Fozia Saeed, Zahid Hussain, Abdul Waheed, Arafat Abdel Hamed Abdel Latef, Nijat Imin

**Affiliations:** 1 Faculty of Engineering, Computing and Science, Western Sydney University, Penrith, NSW 2751, Australia; 2 Research School of Biology, Australian National University, Acton, ACT 2601, Australia; 3 Atta-ur-Rahman School of Applied Biosciences, National University of Sciences and Technology, Islamabad 44000, Pakistan; 4 Institute of Molecular Biology and Biotechnology, Bahauddin Zakariya University, Multan 60800, Pakistan; 5 Agricultural Genomics Institute at Shenzhen, Chinese Academy of Agricultural Sciences (AGIS-CAAS), Shenzhen 518120, China; 6 Botany and Microbiology Department, Faculty of Science, Qena University, Qena 83523, Egypt

**Keywords:** abiotic stress tolerance, hormonal crosstalk, plant signalling networks, plant peptide hormones (CEPs), phytohormone interactions, stress signalling pathways

## Abstract

Plants are constantly exposed to a wide range of abiotic stresses that have significant negative impacts on growth and yield. Plant acclimation to these stresses is governed by integrated classical phytohormone and plant peptide hormone signalling networks that control the ability of a plant to survive and adapt to extreme environments. Classical phytohormones, including abscisic acid, auxins, gibberellins, cytokinins, jasmonates, salicylic acid, brassinosteroids, and the recently recognised phytomelatonin, act in concert with peptide-based plant hormones, among which C-terminally encoded peptides (CEPs) play prominent roles in coordinating stress perception, signal transduction, and adaptive responses throughout the plant. These integrated networks control stomatal behaviour, photosynthesis, osmolyte and antioxidant levels, root architecture, and energy metabolism, thereby helping plants maintain homeostasis and optimise survival while sustaining minimal growth under unfavourable conditions. Under stressful conditions, these networks do not operate in isolation but form highly dynamic, context-dependent regulatory circuits in which each physiological process is simultaneously regulated by multiple hormones acting through convergent and overlapping signalling pathways. Phytomelatonin has emerged as a particularly important integrative node within these networks, functioning both as a potent direct antioxidant through sequential ROS-scavenging catabolite cascades and as a bidirectional regulator of classical phytohormone signalling under diverse abiotic stresses. New technologies in the fields of transcriptomics, proteomics, phosphoproteomics, metabolomics, and systems biology have provided new information on the dynamic relationships between classical phytohormones and plant peptide hormones, revealing candidate regulatory nodes and transcription factor networks that mediate stress adaptation at molecular, biochemical, and physiological levels. However, it is important to distinguish between correlative associations identified through omics profiling and causal regulatory relationships validated through rigorous genetic and biochemical experimentation, as most omics-derived candidates remain to be functionally established. Empirical studies demonstrate how these networks can be used to improve crops by increasing stress tolerance through modulating classical phytohormone and plant peptide hormone signalling, including through exogenous phytomelatonin application, CRISPR-mediated hormone pathway editing, and CEP pathway manipulation, to produce resilient cultivars without reducing yields. Although these advances represent significant progress, challenges remain, including the inherent complexity and redundancy of the networks, context-dependence and severity-dependence of hormonal responses, the persistence of a significant translational gap between laboratory findings and field application, and incomplete mechanistic understanding of peptide hormone roles under combined stress conditions. Addressing these challenges will require integrative multi-omics approaches, higher-order computational modelling, and rigorous field-based functional validation alongside emerging tools such as synthetic biology and precision breeding.

## 1. Introduction

**Figure 1:**
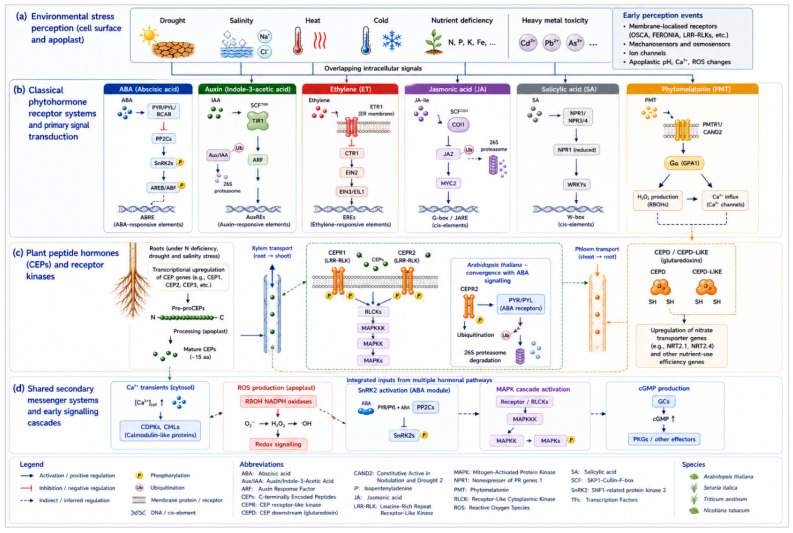
Schematic overview of the primary hormone and plant peptide hormone receptor systems and early signal transduction events triggered by abiotic stress in plants. (**a**) Environmental stresses, including drought, salinity, heat, cold, nutrient deficiency, and heavy metal toxicity, are perceived at the cell surface and apoplast, generating overlapping intracellular signals that activate multiple hormonal signalling pathways simultaneously. (**b**) Classical phytohormone receptors mediate initial signal perception across multiple pathways. ABA is recognised by *PYR/PYL/RCAR* soluble receptors in the cytosol, which inhibit PP2C phosphatases to release SnRK2 kinase activity. Auxin (IAA) binds the *SCF-TIR1* co-receptor complex, promoting *Aux/IAA* repressor degradation and *ARF* transcription factor activation. Ethylene activates ER-localised *ETR1* receptors, leading to *CTR1* inactivation, *EIN2* activation, and *EIN3/EIL1* transcription factor stabilisation. Jasmonates are perceived via the *COI1* F-box receptor in the *SCF-COI1* complex, triggering *JAZ* repressor degradation and *MYC2* activation. Salicylic acid is perceived through *NPR1* and related co-receptors, activating WRKY transcription factor-dependent defence gene expression. Phytomelatonin (PMT) is perceived by the membrane-localised *PMTR1/CAND2* receptor, which signals through the heterotrimeric G protein alpha subunit *GPA1* to trigger downstream H_2_O_2_ production and Ca^2+^ influx cascades. (**c**) Plant peptide hormones, including C-terminally encoded peptides (CEPs), produced in roots under nitrogen deficiency, drought, and salinity stress, are transported via the xylem to shoots where they are recognised by leucine-rich repeat receptor-like kinases *CEPR1* and *CEPR2*, which are expressed predominantly in phloem companion cells. *CEPR2* additionally directly phosphorylates *PYR/PYL* ABA receptors, constituting a mechanistic convergence node between peptide and classical hormone signalling. (**d**) Receptor activation initiates secondary messenger cascades that are shared across multiple hormonal pathways: cytosolic Ca^2+^ transients decoded by CDPKs and calmodulins, apoplastic ROS production by *RBOH* NADPH oxidases, activation of SnRK2 kinases following PP2C phosphatase inhibition by ABA-bound *PYR/PYL* receptors, MAPK cascade activation downstream of multiple receptor systems, and cGMP production contributing to guard cell and stress signalling. Each of these secondary messengers integrates inputs from multiple hormonal pathways simultaneously rather than being exclusively assigned to a single hormone axis. Filled arrows denote activation; blunt-headed lines denote inhibition or repression. Colour coding: blue, ABA pathway; orange, CEP and plant peptide hormone signalling; green, auxin pathway; red, ROS signalling; purple, downstream kinase cascades; yellow, PMT/*PMTR1* signalling; grey, SA/*NPR1* pathway. Dashed lines indicate indirect or inferred regulatory connections.

Plants are sessile organisms subject to a broad array of adverse environmental perturbations that can compromise their growth, development, and productivity. Among these perturbations, non-living environmental stresses collectively termed abiotic stresses represent some of the most severe constraints on plant performance and global food security. Abiotic stress constitutes a heterogeneous group of detrimental environmental parameters, including drought, salinity, heat, cold, nutrient depletion, and heavy metal toxicity, all of which disturb plant physiological homeostasis and alter cellular metabolism in ways that compromise normal growth and development [1]. Despite their distinct physical origins, many abiotic stresses converge on shared physiological and molecular mechanisms, including osmotic imbalance, oxidative stress, membrane disruption, and nutrient dysregulation, generating overlapping cellular responses [1,2]. The interaction of these stressors gives rise to complex signalling networks that ultimately dictate plant survival and adaptive capacity. Phytohormones comprise a diverse group of low-abundance signalling molecules which are necessary in the perception and control of plant stress responses, or in the coordination of plant growth and adaptive events. The classical phytohormones which are widely involved in stress resilience include abscisic acid (ABA), ethylene (ET), auxins, gibberellins (GAs), cytokinins (CKs), jasmonic acid (JA), salicylic acid (SA), brassinosteroids (BRs), and strigolactones (SLs). These phytohormones act in a coordinated and context-dependent manner, forming the regulatory backbone of plant stress adaptation [3]. Such crosstalk can be positive or negative, with interactions that regulate transcriptional outputs, signal transduction strength, and physiological responses (Figure 1). Examples of such crosstalk include ABA-mediated reduction in stomatal conductance under water deficit, which is modulated by ethylene through attenuation of *SnRK2.6* activity, the synergistic interaction between JA and SA in coordinating transcriptional responses to combined biotic and abiotic stresses, and the regulation of root system architecture by auxin and phytomelatonin (PMT), which act in a partially overlapping yet mechanistically distinct manner. Auxin promotes both primary and lateral root growth through polar transport, while PMT stimulates lateral and adventitious root formation and suppresses or minimally affects primary root elongation through modulation of auxin biosynthesis and *PIN*-mediated polar auxin transport [4,5,6]. Recent studies have revealed that plant peptide hormones play important systemic roles in nutrient signalling and stress responses, expanding the known repertoire of plant hormonal regulators beyond classical phytohormones. Among these, C-terminally encoded peptides (CEPs) constitute a family of plant peptide hormones responsible for long-distance communication between root and shoot tissues. CEPs are predominantly expressed in roots in response to nutrient deficiency, particularly nitrogen limitation, though their expression has been documented in multiple tissues including shoots and vascular tissue under various abiotic and biotic conditions [7,8]. Root-derived CEPs are transported through the xylem to aerial parts where they bind specific receptors, including *CEPR1*, to coordinate adaptive changes in nutrient uptake and root architectural development [9]. These hierarchical networks integrate hormone perception, secondary messengers, transcription factors, and post-translational regulation to implement coordinated responses at molecular, cellular, and physiological levels, enabling plants to balance growth and defence during dynamic environmental challenges [2] (Figure 1).

## 2. Overview of Classical Phytohormones and Peptide Plant Hormones in Abiotic Stress

### 2.1. Classical Hormones

Classical phytohormones regulating abiotic stress responses encompass two broad structural categories, namely classical phytohormones and peptide plant hormones. Among the classical phytohormones, the principal regulators of stress responses include auxins (indolic compounds of which indole-3-acetic acid (IAA) is the principal active form), GAs (diterpenoid acids), CKs (adenine derivatives with isoprenoid or aromatic side chains), ABA (a sesquiterpenoid derived from carotenoid cleavage), jasmonates (cyclopentanone derivatives of linolenic acid), brassinosteroids (polyhydroxylated steroidal lactones), salicylic acid (a phenolic compound), phytomelatonin (PMT; an indoleamine derived from tryptophan), strigolactones (carotenoid-derived terpenoid lactones), and karrikins (smoke-derived butenolides), all of which interact with fundamental processes inherent to plant responses to abiotic stresses [10,11]. The distinction between these molecules is structural and biosynthetic rather than functional, as all contribute to stress perception, signalling, and adaptive responses. The stress-specific roles of PMT and its interactions with classical phytohormones under abiotic stress are discussed in detail in Section 4.8. Strigolactones regulate shoot branching and symbiotic signalling and have recently been implicated in drought and phosphate deficiency responses. Phytomelatonin (PMT; N-acetyl-5-methoxytryptamine) is increasingly recognised as a bona fide plant hormone with widespread roles in abiotic stress tolerance, antioxidant defence, and phytohormone crosstalk. PMT is synthesised in plants from tryptophan through a conserved multi-step pathway involving tryptophan decarboxylase (*TDC*), tryptamine 5-hydroxylase (*T5H*), serotonin N-acyltransferase (*SNAT*), and N-acetylserotonin methyltransferase (*ASMT*) and/or caffeic acid 3-O-methyltransferase (*COMT*) [11,12]. A landmark advance in 2018 was the identification of the first plant melatonin receptor, *PMTR1* (also designated *CAND2*), a candidate G protein-coupled receptor in *Arabidopsis* that mediates PMT perception through the heterotrimeric G protein α-subunit *GPA1*, triggering downstream H_2_O_2_ and Ca^2+^ signalling cascades [13]. Loss-of-function *cand2* mutants display impaired stomatal closure and reduced osmotic stress tolerance, confirming that *PMTR1*-dependent signalling is required for PMT-mediated stress adaptation [14]. PMT exerts its protective effects through two complementary pathways such as a direct antioxidant pathway in which PMT and its catabolites chemically scavenge ROS, and an indirect signalling pathway in which PMT activates MAPK cascades and modulates the expression of stress-responsive genes through *PMTR1*-mediated signalling [11,15]. Karrikins, smoke-derived butenolides, regulate seed germination and early seedling development under stress [10]. Salicylic acid (SA), though primarily associated with biotic stress responses, also contributes to abiotic stress tolerance by modulating antioxidant enzyme activity, regulating hydrogen peroxide levels, and interacting synergistically with ABA and JA signalling networks under combined osmotic and oxidative stress conditions [16].

In *Arabidopsis*, the mechanistic basis of auxin action under abiotic stress is best established through the *TIR1/AFB* receptor system, in which auxin binding to the *TIR1* F-box protein promotes ubiquitination and 26S proteasome-mediated degradation of *Aux/IAA* transcriptional repressors, thereby releasing *ARF* transcription factors to drive stress-responsive gene expression [17]. Polar auxin transport is maintained by PIN-FORMED (PIN) and PIN-LIKE (PILS) efflux carriers, whose phosphorylation-dependent subcellular localisation determines the spatial distribution of auxin in roots under stress [18]. In *Arabidopsis*, low salinity suppresses lateral root elongation while stimulating lateral root number in *axr1*, *axr4*, and *tir1* receptor mutants, whereas high salinity completely suppresses primary root elongation, demonstrating that auxin sensitivity thresholds determine the architectural response to salt stress [19,20]. Comparable auxin transport-dependent root architectural responses to drought and salinity have since been documented in rice, maize, and tomato, where ABA-mediated suppression of PIN protein abundance similarly redirects root growth patterns under osmotic stress [21].

Gibberellins play a role in regulating various developmental processes, such as cell elongation, seed germination, internodal growth, flowering, and fruit development [22]. In *Arabidopsis*, the regulatory axis connecting GA and stress tolerance centres on DELLA proteins, which are destabilised by GA-mediated activation of the *GID1* receptor-SCF^*SLY1* ubiquitin ligase complex under favourable conditions, allowing GA-driven growth. Under dehydration and salinity stress, reduced GA accumulation stabilises DELLA proteins, which in turn activate ROS-scavenging gene networks and represses GA-dependent growth, thereby redirecting metabolic resources towards stress protection [23]. *Arabidopsis* quadruple *della* loss-of-function mutants show reduced ROS accumulation under salinity stress, accompanied by upregulation of antioxidant biosynthesis genes, providing genetic evidence for the DELLA-dependent oxidative stress protection mechanism [23]. This *Arabidopsis* model is conserved in other species such as in maize, reduced GA accumulation under dehydration increases DELLA activity and ROS scavenging capacity, and in rice, the *SUB1A* gene acts through the DELLA proteins (*SLR1* and *SLRL1*) to suppress ROS production under submergence, reducing oxidative damage and improving survival [24]. Cytokinins promote cell division, shoot growth, and photosynthetic capacity under favourable conditions and under mild stress. Under mild stress, CK signalling remains active and contributes to growth maintenance and metabolic homeostasis, whereas under severe or prolonged stress, the CK signalling pathway is actively downregulated, redirecting metabolic resources from growth toward stress acclimation responses. The role of CKs under stress is therefore strongly dependent on stress severity, tissue type, and developmental stage, as discussed in detail in Section 4.3. ABA is a central mediator of stress adaptation, regulating seed dormancy, stomatal closure, and water-use efficiency. Under water limitation, ABA biosynthesis is enhanced in roots, and the hormone is translocated to leaves, where it triggers guard cell signalling to limit transpiration and conserve water [25].

### 2.2. Plant Peptide Hormones: CEPs and Others

C-terminally encoded peptides (CEPs) are a family of post-translationally modified plant peptide hormones conserved across vascular land plants. The name reflects the genomic organisation of their precursor proteins. The biologically active peptide domain is encoded at the C-terminal end of the precursor open reading frame, distinguishing this family from other plant peptide hormones whose active domains are N-terminally encoded or centrally located [26]. CEP precursor proteins possess an N-terminal secretion signal peptide that directs them to the secretory pathway, followed by a variable domain and one or more conserved CEP domains of approximately 15 amino acids in length. Proteolytic processing of the precursor in the apoplast releases the mature 15-amino acid CEP [26,27]. The mature CEP is characterised by the presence of conserved proline residues typically at positions 4 and 11 which undergo post-translational hydroxylation to form hydroxyproline, and in some cases further arabinosylation at position 11 [7,28]. This hydroxylation and arabinosylation modifications are essential for full biological activity and receptor recognition, as demonstrated by NMR studies showing that hydroxyproline residues influence peptide flexibility and *CEPR1/CEPR2* receptor binding affinity [7,29]. More than 900 CEP genes have been identified across plant species to date, reflecting the evolutionary conservation and diversification of this peptide hormone family [30].

**Figure 2:**
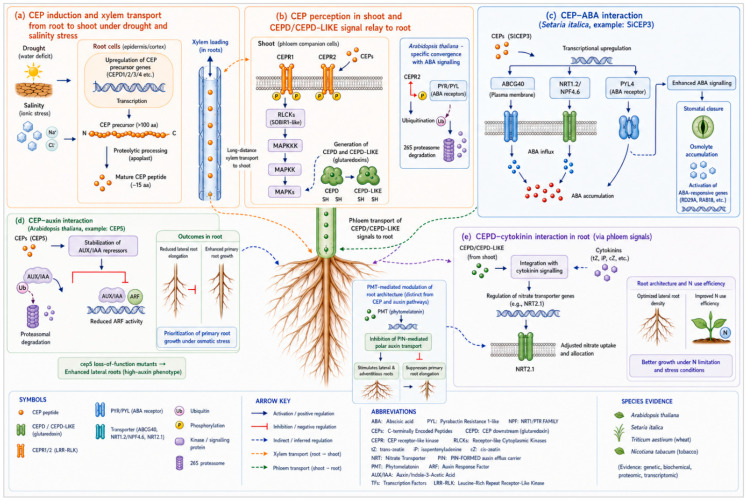
Regulatory network depicting the mechanistic interactions between C-terminally encoded peptides (CEPs) and classical phytohormones under drought and salinity stress conditions, based on genetic, biochemical, and proteomic evidence from *Arabidopsis thaliana*, *Setaria italica*, wheat, and tobacco. (**a**) Under water-deficit or salt stress, CEP precursor genes are transcriptionally upregulated in root cells, producing mature CEPs of approximately 15 amino acids that undergo apoplastic proteolytic processing and are loaded into the xylem for long-distance transport to the shoot [26,27]. (**b**) In shoot tissues, mature CEPs are perceived by membrane-localised leucine-rich repeat receptor-like kinases *CEPR1* and *CEPR2*, which are expressed predominantly in phloem companion cells, triggering downstream phosphorylation cascades that generate *CEPD* and *CEPD-LIKE* glutaredoxin signals that are translocated back to the root via the phloem to upregulate nitrate transporter gene expression, establishing a bidirectional long-distance signalling loop [34,35]. In *Arabidopsis*, *CEPR2* additionally directly phosphorylates ABA receptors of the *PYR/PYL* family, promoting their degradation and modulating ABA sensitivity under stress, thereby constituting a mechanistic convergence node between CEP signalling and classical ABA signalling [36]. (**c**) CEP-ABA interaction: in *Setaria italica*, the *SiCEP3* enhances ABA accumulation by transcriptionally upregulating the expression of plasma membrane-localised ABA influx transporter genes *ABCG40* and *NRT1.2/NPF4.6*, as well as the ABA receptor gene *PYL4*, thereby increasing cellular ABA uptake capacity and amplifying downstream ABA signalling, resulting in stomatal closure, osmolyte accumulation, and activation of ABA-responsive genes including *RD29A* and *RAB18* [37]. (**d**) CEP-auxin interaction: in *Arabidopsis*, the *CEP5* stabilises *AUX/IAA* transcriptional repressors, attenuating ARF-dependent auxin responses and reducing lateral root elongation, thereby prioritising primary root growth under osmotic stress, as confirmed by loss-of-function *cep5* mutants displaying enhanced lateral root formation phenocopying high-auxin conditions [38]. (**e**) CEP-cytokinin interaction: *CEPD* glutaredoxin signals returning to roots through the phloem integrate CEP and cytokinin signalling pathways to adjust nitrogen transporter expression, particularly *NRT2.1*, and regulate root architecture in response to nitrogen availability and environmental stress [39]. Note that phytomelatonin (PMT) additionally modulates root architecture through overlapping yet mechanistically distinct pathways from CEP and auxin, stimulating lateral and adventitious root formation while suppressing primary root elongation through inhibition of PIN-mediated polar auxin transport [5,6], as discussed in Section 4.8. Synergistic interactions are depicted by blue arrows; antagonistic interactions, by red blunt-headed lines; dashed lines indicate indirect or inferred regulatory connections supported by transcriptomic or genetic but not direct biochemical evidence.

Recent studies in crop species, such as wheat and tomato, indicate that CEPs increase drought and salt tolerance. The crosstalk of CEP with other hormones and their impact on the plant under stressed environmental condition showed in Figure 2. Exogenously applied wheat peptide *TaCEP1D* has been reported to overcome the effects of drought when sprayed onto the wheat foliage. Consequently, more work is needed to characterize the specific receptors and signalling pathways involved in the regulation of drought- and salt-stress tolerance mediated by CEPs [31,32]. Emerging evidence emphasizes the key role of small plant peptide hormones in coordinating plant responses to drought and other abiotic stresses, though the mechanistic understanding of many peptide classes remains incomplete. Several such peptides have been identified as contributors to increased plant drought tolerance. Notably, the rice-derived peptide *OsS1Fa1* has been linked to enhanced drought resistance when heterologously overexpressed in *Arabidopsis thaliana*. Transgenic analyses further demonstrate that *S1Fa* peptides confer tolerance to various abiotic stresses in other species, including maize and Chinese cabbage. Plant elicitor peptides (PIPs) are another class of small peptides that are involved in the integration of stress signalling pathways. The *PIP3* peptide, produced in response to pathogen-associated molecular patterns (PAMPs), is detected by the receptor-like kinase RLK7, which then activates mitogen-activated protein kinases (*MPK3* and *MPK6*). While PIP-RLK7 signalling cascade is thoroughly described in plant immune responses, new evidence shows a role for this pathway in abiotic stress adaptation, including salt stress, possibly through modulation of stress-related signalling cascades and ionic homeostasis [33].

Temperature stress is also an additional risk factor for plant viability, but the role of small peptides in temperature-stress responses remains poorly understood. Low-temperature stress has been shown to affect membrane integrity, ROS homeostasis, osmolyte accumulation, and antioxidant capacity. Beyond developmental roles, peptide products of primary microRNA transcripts, termed microprotein-encoded peptides (miPEPs), have been found to mediate tolerance to cold stress. In grapevine, miPEPs such as *vvi*-*miPEP172b* and *vvi-miPEP3635b* are cold-responsive peptides encoded within pri-miRNA transcripts that enhance transcription of their corresponding miRNA genes, thereby amplifying cold-stress-responsive miRNA accumulation. Conversely, through high temperature conditions, the *CLE45* peptide, which is produced in the stigma and extends its expression domain into the transmitting tract, may be involved in the maintenance of the pollen tube and thus a reproductive adaptation to heat stress [6].

## 3. Hormonal Signalling Pathways in Stress Perception

### 3.1. Signal Perception and Transduction

**Figure 3:**
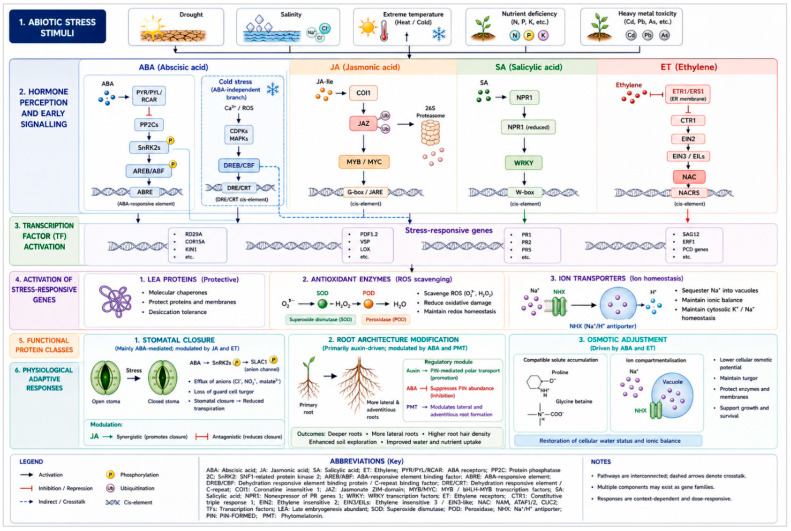
Hormone-mediated signal transduction and transcriptional regulatory cascades governing plant adaptive responses to abiotic stress. Abiotic stress stimuli, including drought, salinity, extreme temperatures (heat and cold), nutrient deficiency, and heavy metal toxicity, activate coordinated responses through multiple phytohormone signalling pathways. While almost all classical phytohormones contribute to abiotic stress adaptation, this figure illustrates four well-characterised hormone signalling cascades, namely abscisic acid (ABA), jasmonic acid (JA), salicylic acid (SA), and ethylene (ET), for which the receptor-to-transcription factor regulatory architecture has been most precisely resolved at the molecular level. The roles of additional phytohormones including auxin, gibberellins, cytokinins, brassinosteroids, strigolactones, and phytomelatonin in abiotic stress responses are integrated into the physiological adaptive responses shown in row 6 and are discussed in detail in Section 2.1, Section 4.1, Section 4.2, Section 4.3, Section 4.4, Section 4.5, Section 4.6, Section 4.7, Section 4.8, Section 5.1, Section 5.2, Section 5.3, Section 5.4 and Section 5.5. Row 2 depicts hormone perception and early signalling events for each pathway. ABA is perceived by cytosolic *PYR/PYL/RCAR* soluble receptors, which inhibit PP2C phosphatases and thereby activate SnRK2 kinases through autophosphorylation, leading to phosphorylation and activation of *AREB/ABF* transcription factors that bind ABA-responsive elements (*ABREs*) in the promoters of drought and osmotic stress-responsive genes. Under cold stress, an overlapping yet partially ABA-independent pathway additionally engages *DREB/CBF* transcription factors through Ca^2+^, and ROS signals decoded by *CDPKs* and MAPKs, leading to binding of DRE/CRT cis-elements and activation of cold-responsive genes. JA-Ile, the biologically active jasmonate conjugate, is perceived by the *COI1* F-box protein in the *SCF-COI1* ubiquitin ligase complex, promoting ubiquitination and 26S proteasome-mediated degradation of JAZ repressor proteins, thereby releasing *MYB/MYC* transcription factors to bind G-box and JARE cis-elements and activate defence and stress-adaptive gene expression. SA is perceived through the *NPR1* co-receptor, which in its reduced monomeric form translocate to the nucleus to activate *WRKY* transcription factors that bind W-box cis-elements and coordinate both biotic and abiotic stress-responsive gene expression. ET is perceived by *ETR1/ERS1* receptors at the endoplasmic reticulum membrane, which in the absence of ethylene constitutively activate *CTR1* to repress downstream signalling. Ethylene binding inactivates *ETR1/ERS1*, relieving *CTR1* repression and allowing *EIN2* activation, which stabilises *EIN3/EIL* transcription factors that engage *NAC* transcription factors binding NACRS cis-elements to modulate senescence and stress tolerance programmes. Row 3 shows the transcription factor activation layer for each pathway, with representative stress-responsive target genes listed for each hormone axis. ABA-AREB/ABF activation induces *RD29A*, *COR15A*, *KIN1*, and related osmotic stress genes. JA-MYB/MYC activation induces *PDF1.2*, *VSP*, *LOX*, and related defence metabolite genes. SA-WRKY activation induces *PR1*, *PR2*, *PR5*, and related pathogenesis-related genes. ET-NAC activation induces *SAG12*, *ERF1*, *PCD* genes, and related senescence and tolerance genes. Row 4 shows the activation of stress-responsive genes encoding three principal functional protein classes. First, LEA proteins act as molecular chaperones that protect proteins and membranes and confer desiccation tolerance. Second, antioxidant enzymes including superoxide dismutase (SOD) and peroxidase (POD) act sequentially to scavenge ROS, with SOD dismutating superoxide radical anions to hydrogen peroxide and POD further reducing hydrogen peroxide to water, thereby reducing oxidative damage and maintaining redox homeostasis. Third, NHX ion transporters sequester excess Na^+^ into vacuoles under salinity stress, maintain ionic balance, and support cytosolic K^+^/Na^+^ homeostasis. Row 6 shows three coordinated physiological adaptive responses that integrate inputs from multiple phytohormone pathways simultaneously, not solely from the four axes depicted above. First, stomatal closure is primarily mediated by ABA acting through SnRK2-dependent phosphorylation of the *SLAC1* anion channel, driving efflux of anions including Cl^−^, NO_3_^−^, and malate^2−^, loss of guard cell turgor, and reduced transpiration. JA acts synergistically with ABA to promote closure through shared guard cell signalling components, while ET antagonises ABA-induced closure by promoting ABI1 and ABI2 PP2C phosphatase stability. Second, root architecture modification is primarily driven by auxin through PIN-mediated polar transport promoting lateral and adventitious root formation, while ABA suppresses PIN protein abundance at the plasma membrane through endosomal trafficking to inhibit lateral root initiation and sustain primary root elongation, and phytomelatonin (PMT) modulates lateral and adventitious root formation through inhibition of PIN-mediated polar auxin transport. Together these hormonal inputs result in deeper roots, more lateral roots, higher root hair density, enhanced soil exploration, and improved water and nutrient uptake. Third, osmotic adjustment is driven by ABA-dependent and ET-dependent accumulation of compatible solutes including proline and glycine betaine, which lower cellular osmotic potential to maintain turgor, protect enzymes and membranes, and support growth and survival, alongside ion compartmentalisation through NHX-mediated Na^+^ sequestration to restore cellular water status and ionic balance. Pathways are interconnected and responses are context-dependent and dose-responsive. The four hormone signalling cascades depicted in row 2 were selected on the basis of mechanistic resolution at the receptor-to-transcription factor level and do not imply greater importance than auxin, GA, CK, BR, SL, or PMT, whose roles in abiotic stress adaptation are equally significant and are discussed throughout the text.

Plants’ ability to survive and adapt to abiotic stress depends on highly efficient architectures for signal perception and transduction (Figure 3). Hormonal signalling cascades serve as central integrators of environmental information, translating external stress cues into specific intracellular molecular responses. Abiotic stress perception is triggered by specific hormone receptors, and downstream signalling pathways are activated through secondary messengers and reversible post-translational modifications, ultimately leading to transcriptional reprogramming and physiological adjustments. ABA perception is mediated by a family of soluble receptors, of which *PYRABACTIN RESISTANCE* (*PYR*)/*PYR1-LIKE* (*PYL*)/regulatory components of ABA receptors (*RCAR*) are members. Structural and biochemical investigations show that ABA binding to *PYR/PYL* receptors promotes interaction with clade IIC type 2C protein phosphatases (*PP2Cs*) and inhibits their activity, which frees SnRK2 kinases from repression [40]. Consequently, ABA perception is directly linked to rapid phosphorylation cascades to regulate stomatal closure, osmoprotectant synthesis, and stress-responsive gene expression. Auxin perception via the *TIR1/AFB-Aux/IAA* system (described in Section 2.1) modulates adaptive root responses under stress, including altered lateral root formation and stress-induced morphogenesis. Ethylene perception involves a separate receptor system that is localized to the endoplasmic reticulum membrane. ETHYLENE RECEPTOR (ETR), e.g., *ETR1*, are negative regulators of ethylene signalling in the absence of the hormone. The binding of ethylene inactivates these receptors, thereby relieving inhibition of the Raf-like kinase CONSTITUTIVE TRIPLE RESPONSE1 (*CTR1*), which acts to down-regulate the pathway [41,42]. Inactivation of *CTR1* allows the activation of ETHYLENE INSENSITIVE 2 (*EIN2*), which then stabilizes the *EIN3/EIL* transcription factors that direct stress-responsive gene expression.

CEP perception in *Arabidopsis* is mediated by the leucine-rich repeat receptor-like kinases *CEPR1* and *CEPR2*, which are expressed predominantly in shoot phloem companion cells and function as the primary receptors for CEPs transported from roots through the xylem under nitrogen limitation [9,34]. Upon CEP binding, *CEPR1* and *CEPR2* activate downstream signalling that induces the production of *CEPD* and *CEPD*-*LIKE* glutaredoxin signals in the shoot, which subsequently migrate back to roots through the phloem to upregulate nitrate transporter gene expression, establishing a bidirectional long-distance signalling loop [35] (Figure 2). Recent work in *Arabidopsis* has further revealed that *CEPR2* can directly phosphorylate ABA receptors of the *PYR/PYL* family, promoting their degradation and modulating ABA sensitivity under stress conditions, thereby identifying a mechanistic convergence node between peptide and classical hormone signalling [36] (Figure 2). Whether this *CEPR2-PYL* phosphorylation axis is conserved in crop species such as wheat and rice, where CEP-mediated drought and salt tolerance have been reported, remains an important open question for future investigation [31,32]. Following receptor activation, the signal is amplified through secondary messengers such as Ca^2+^, ROS, and cyclic guanosine monophosphate (cGMP). Transient cytosolic Ca^2+^ elevations are decoded by calcium-binding proteins including calmodulins, calcineurin-B-like proteins, and CDPKs, which translate stress-specific calcium signatures encoding information about stimulus intensity and duration into distinct phosphorylation cascades, thereby conferring signal specificity [43,44]. Reactive oxygen species, once considered cytotoxic metabolic by-products, have important signalling roles during stress perception. A group of enzymes known as NADPH oxidases produce apoplastic ROS that function as systemic signals and modulate the gating of plasma membrane ion channels, including those controlling guard cell turgor [45].

### 3.2. Downstream Signalling Components

Following initial hormone perception and the generation of second messengers such as Ca^2+^ and ROS, plant cells engage a complex network of downstream signalling components that transduce and amplify stress signals to effectors in the cytoplasm and nucleus. At the centre of this signalling relay is the protein kinases and phosphatases which coordinate switches dependent on phosphorylation and are how multiple hormonal and environmental signals can be integrated to adjust stress-adaptation mechanisms. Activated by upstream ABA perception through the *PYR/PYL-PP2C* module (Section 3.1), the subclass III SnRK2 group (*SnRK2.2*, *SnRK2.3*, and *SnRK2.6/OST1*) undergoes autophosphorylation and activation [46]. The phosphorylation of a range of downstream targets including the transcription factors *AREB/ABF* and the anion channel *SLAC1* by the activated SnRK2s results in the stomatal closure and expression of stress-responsive genes. Altogether, ABA-dependent receptor phosphorylation is complemented by the upstream MAP kinase kinase kinases (M3Ks) being able to respond to osmotic signals, suggesting that the ABA-independent responses of SnRK2s lead to the same kinases to guarantee efficient propagation of the stress signal [47]. Mitogen-activated protein kinases (MAPKs) are an essential group of downstream signal transducer enzymes that send hormones and environmental stress signals through three-level phosphorylation cascades that include MAP kinase kinase kinases (MAPKKKs), MAP kinase kinases (MAPKKs), and MAPKs themselves [48]. In the abiotic stress scenario, different MAPK modules are stimulated by drought, salinity and cold, thus, playing a role in abscisic acid (ABA)-dependent and -independent signalling crosstalk. One of the representative examples is the *Arabidopsis MAPKKK17/18* -*MKK3* -*MPK1/2/7/14* cascade induced by ABA and mediating stomatal regulation and transcriptional changes in response to ABA during drought stress. MAPKs are mechanistically integrated with ROS and Ca^2+^ signalling through shared upstream activators and shared downstream transcription factor targets [49]. Negative regulation is governed by clade A *PP2Cs*, including *ABI1* and *ABI2*, which are inhibited by ABA-bound *PYR/PYL* receptors; this kinase--phosphatase balance dictates the magnitude and duration of SnRK2-mediated stress responses [50]. Finally, downstream signalling mediators are centrally sorted around the transcriptional regulators, such as basic leucine zipper (bZIP)-type proteins in the form of ABA-responsive element binding proteins (AREBs/ABF) and dehydration-responsive element binding proteins (DREBs), and NAC domain proteins among other stress-sensitive proteins. This phosphorylation by kinases increases their DNA-binding capacity and controls the transcription of ABA responsive and stress inducing genes and is therefore a direct connection of early kinase cascades to genome-wide transcriptional reprogramming, which is required to survive abiotic stress. The phosphorylation of these transcription factors is regulated by a complex of kinases (SnRK2s, CDPKs, and MAPKs) and phosphatases which constitute a tightly regulated network governing stress-adaptive response [51].

### 3.3. Transcriptional Regulation

Transcriptional regulation constitutes one of the most important levels of hormonal signalling that leads to the response of abiotic stress and is characterized by the integration of the precedent perception trajectory with the adaptive program of gene expression. When stress is sensed, these hormone-dependent signal transduction pathways target specific transcription factors (TFs), and the reaction of these groups of proteins coordinates massive reconfiguration of stress-responsive transcription. The best-studied hormonal pathway for stress-induced transcriptional regulation involves ABA. Through the *PYR/PYL-PP2C-SnRK2* cascade (Section 3.1), ABA activates *AREB/ABF* transcription factors, which bind ABA-responsive elements (*ABREs*) in promoter regions of regulated genes [52]. AREB/ABFs control some of the most important drought- and salinity-inducible genes, including *RD29B*, *RAB18*, and LEA family members, thereby mediating osmotic adjustment and cell defence. The genetic evidence supporting the functional role of SnRK2-dependent transcriptional activation in stress tolerance is that, in *Arabidopsis*, triple loss-of-function mutations in *SnRK2.2/SnRK2.3/SnRK2.6* resulted in strong ABA insensitivity and impaired drought tolerance [53]. Parallel to ABA-dependent processes, ABA-independent transcriptional regulation is enabled by DREB (dehydration-responsive element-binding) proteins, which include the *DREB1/CBF* and *DREB2* sub-classes. These TFs are DRE/CRT cis-element binders and control cold- and dehydration-regulated genes in the absence of ABA accumulation. *DREB1A* overexpression has been reported to increase freezing and drought tolerance, and hence the significance of such a transcriptional module [54]. Notably, ABA-dependent and ABA-independent pathways are cross-regulated and integrated at the transcriptional level, where shared transcription factors bind to overlapping promoter elements of stress-responsive genes, enabling coordinated yet flexible regulation of the stress transcriptome. This convergence at the level of transcription factor–promoter interactions constitute a key mechanism of pathway integration within the stress signalling network [52]. There are other hormone-controlled TF families that enhance abiotic stress adaptation. Examples of these include *NAC* transcription factors, such as *SNAC1* in rice, which are involved in increasing drought tolerance through stomatal control and root architecture [55]. MYB and WRKY proteins integrate ABA, jasmonate, and salicylic acid signalling in response to osmotic and oxidative stress [56]. The result of ethylene signalling is the activation of *EIN3/EIL* TFs [57], which control stress-responsive genes and interact with ABA-mediated signals, suggesting transcriptional convergence among hormonal networks. Hormone-responsive gene expression is further tuned by chromatin remodelling, histone modifications, and transcriptional coactivators, thereby allowing rapid yet reversible responses to changing environmental signals. Transcriptional regulation, therefore, represents a key integrative point in hormonal crosstalk, where a variety of signalling inputs are converted into a coordinated physiological response [57].

### 3.4. Post-Translational Modifications (PTMs)

Post-translational modifications (PTMs) constitute an important layer of regulation in plant stress signalling and enable rapid, reversible modulation of protein activity after translation. The addition of specific chemical groups to amino acid residues through covalent bonds influence protein stability, subcellular localisation, interaction capacity, and enzymatic function without requiring changes in gene expression, thereby constituting a rapid mechanism for plants to adapt to diverse environmental stresses such as drought, salinity, heat, and cold [58]. Phosphorylation is one of the best-characterized PTMs of plant hormone signalling pathways. Protein kinases and phosphatases dynamically modulate the phosphorylation states of signalling components and thereby regulate stress-responsive pathways. For example, phosphorylation of transcription factors and signalling proteins triggers the rapid induction of stress response networks in response to environmental stimuli. In the ABA signalling cascade, phosphorylation events coordinated by SnRK2s (Section 3.2) exemplify how PTMs rapidly activate stress-responsive gene networks without requiring transcriptional changes to signalling components. Ubiquitination is another key PTM in abiotic stress responses, in which the ubiquitin-proteasome system mediates protein turnover. In plant hormone signalling networks, ubiquitination is often important in modulating the abundance of key regulatory proteins and thus fine-tuning hormonal responses to stress. For instance, phosphorylation of certain substrates can lead to the formation of “phosphodegron” motifs, which are recognised by ubiquitin ligases, leading to ubiquitination-mediated degradation and dynamic regulation of stress signalling components [59]. SUMOylation, the conjugation of Small Ubiquitin-like Modifier (SUMO) proteins to target proteins, is also important in regulating plant stress responses. SUMOylation can influence transcription factors and signalling proteins involved in hormone pathways, thereby altering gene expression and cellular responses to external stimuli. For example, SUMOylation of regulatory proteins has been found to affect the activity of transcription factors under heat and drought stress, highlighting its role in stress-responsive signalling cascades [60]. PTMs are also responsible for regulating the activity and stability of hormone receptors themselves. Within the ABA signalling pathway, receptors of the *PYR/PYL/RCAR* family undergo various PTMs, such as phosphorylation and ubiquitination, which regulate their stability, subcellular localisation, and interactions with downstream signalling components. These modifications have allowed plants to fine-tune ABA perception and transduction under stress conditions [61].

## 4. Crosstalk Mechanisms Among Phytohormones and Peptide Hormones

Before discussing individual hormonal crosstalk pairs, it is important to emphasise that each major physiological process regulated during abiotic stress, including stomatal closure, root architecture remodelling, osmolyte accumulation, antioxidant defence, and senescence regulation, is not governed by one or two hormones in isolation but is simultaneously regulated by multiple classical phytohormones acting through convergent and overlapping signalling pathways. For example, stomatal closure is promoted by ABA and jasmonic acid, modulated antagonistically by ethylene, and influenced by auxin, cytokinins, brassinosteroids, and phytomelatonin through interconnected guard cell signalling cascades. Similarly, root architecture is shaped by the coordinated activities of ABA, auxin, cytokinins, ethylene, and PMT, each contributing distinct yet integrated regulatory inputs. The pairwise ABA-centred crosstalk descriptions in the following subsections are therefore a structural simplification adopted for clarity of presentation and should be understood within the broader context of simultaneous multi-hormone regulation of each adaptive process [4,15].

### 4.1. ABA-Auxin Interaction

The crosstalk between ABA and auxin is central to hormonal regulation of plant growth and abiotic stress responses. Under abiotic stress, ABA and auxin act through distinct yet integrated signalling pathways, ABA primarily mediating osmotic and ionic stress responses, and auxin regulating root architecture and developmental plasticity whose crosstalk enables coordinated adaptive responses [62]. In *Arabidopsis*, the mechanistic basis of ABA-auxin interaction under osmotic stress has been most precisely characterised at the level of polar auxin transport regulation. ABA suppresses auxin-responsive *DR5* reporter activity in root tips under osmotic stress, and the ABA signalling component *ABI4* directly reduces *PIN1*-mediated polar auxin transport by binding to the *PIN1* promoter and repressing its transcription, thereby limiting lateral root initiation while sustaining primary root elongation [63]. Additionally, osmotic stress reduces the abundance of *PIN1* and *PIN2* proteins at the plasma membrane through ABA-mediated endosomal trafficking, further restricting auxin gradients in root tissues and contributing to root meristem size reduction and growth inhibition [21,64]. At the transcriptional level in *Arabidopsis*, ABA represses specific ARFs while promoting others, and components of the auxin pathway such as *ARF10* and *ARF16* reciprocally modulate ABA signalling by regulating ABI3 expression, establishing a bidirectional regulatory circuit [65]. The *ABI3-ERF1* regulatory module further integrates ABA-auxin crosstalk in *Arabidopsis* by controlling genes involved in lateral root emergence including *PIN1*, *AUX1*, and *ARF7* [66]. Comparable ABA-auxin interactions regulating root architecture under drought and salinity have since been characterised in rice, maize, and tomato, confirming that the core *Arabidopsis* model of ABA-mediated suppression of PIN-dependent auxin transport is broadly conserved across species, albeit with species-specific transcriptional targets and developmental outcomes [62].

### 4.2. ABA-Ethylene Interaction

In *Arabidopsis*, the molecular antagonism between ABA and ethylene at the stomatal level has been mechanistically resolved through the *OST1/SnRK2.6-SLAC1-ABI1/ABI2* signalling axis. ABA activates *OST1/SnRK2.6*, which phosphorylates the SLAC1 anion channel to drive stomatal closure, while ethylene counteracts this response through EIN3-mediated transcriptional induction of *ABI1* and *ABI2* PP2C phosphatases, which dephosphorylate and inactivate *OST1/SnRK2.6*, thereby attenuating ABA-dependent closure [67]. Genetic evidence from *Arabidopsis* mutants lacking *EIN2* or *EIN3* confirms that ethylene signalling is required for the suppression of ABA-induced stomatal closure under combined stress conditions. Regarding seed germination, ethylene signalling components *EIN2*, *EIN3*, and ethylene-responsive transcription factors in *Arabidopsis* act on ABA biosynthetic and catabolic pathways to promote the transition from dormancy to germination by reducing endogenous ABA levels [68]. A similar *OST1-ABI* regulatory axis has been demonstrated in tomato and rice under combined drought-flooding stress, demonstrating cross-species conservation of this crosstalk module. Furthermore, transcriptomic analyses of *Arabidopsis* mutants defective in both ABA and ethylene signalling reveal cooperative regulation of antioxidants and phenylpropanoid metabolism under combined cold and osmotic stress, a relationship that has also been documented in maize and barley under field-relevant stress combinations [69]. Furthermore, ABA-ethylene crosstalk regulates growth-defense trade-offs under stress conditions. The synergistic action of these two hormones helps plants maximize resource use and maintain physiological balance when faced with environmental stress [70].

### 4.3. ABA-Cytokinin Interaction

ABA-cytokinin (CK) crosstalk is an important regulatory system that modulates plant development and stress acclimation to unfavourable environmental conditions. The role of cytokinins under stress is strongly context-dependent and cannot be characterised categorically. Under well-watered conditions, CKs generally promote stomatal opening and stimulate photosynthetic capacity by maintaining chlorophyll content and RuBisCO activity. Under drought or osmotic stress, endogenous CK levels typically decline in shoots as CK oxidase/dehydrogenase (CKX) activity increases, and this reduction is often associated with stomatal closure and reduced transpiration in coordination with elevated ABA [71,72]. This stress-severity-dependent regulation of CK activity enables plants to maintain growth under manageable stress while prioritising survival under severe conditions. However, the relationship between CK abundance and stress tolerance is not uniformly negative. Stress-inducible elevation of CK levels through *IPT* overexpression driven by senescence- or stress-responsive promoters has been shown to improve drought tolerance in *Arabidopsis*, tobacco, and barley by delaying premature senescence, reducing ROS damage and lipid peroxidation, and redistributing soluble sugars as osmolytes, effects that are beneficial rather than detrimental under stress [73,74]. Furthermore, CK activity in roots and shoots responds to stress differently, reduced CK signalling in roots through *CKX* overexpression enhances root elongation, increases root-to-shoot ratio, and improves soil exploration, thereby conferring drought tolerance through improved water acquisition rather than avoidance [75]. The net effect of CK signalling under stress is therefore tissue-specific and temporally regulated, reflecting the physiological priority of the organ and the severity and duration of the stress. The crosstalk between ABA and CK signalling pathways occurs at the molecular level, and a variety of regulatory components are involved. A two-fold signalling system comprises histidine kinase receptors (AHKs), histidine phosphotransfer proteins (AHPs), and response regulators (ARRs), which mediate CK signalling. There is experimental evidence to indicate that *SNF1*-related protein kinases (SnRK2s), which directly interact with CK response regulators to coordinate stress responses, are components of ABA signalling. Indicatively, *SnRK2.2*, *SnRK2.3*, and *SnRK2.6* phosphorylate type-A response regulator *ARR5*, thereby increasing its stability and suppressing CK signalling in response to drought stress [76]. Further genetic studies have shown that loss-of-function mutations in CK signalling pathway components are often associated with enhanced drought tolerance and reduced shoot growth [76]. In addition to drought responses, there is crosstalk between ABA and CK in seed germination, root architecture, and leaf senescence under stressful conditions. ABA tends to inhibit seed germination and growth in adverse environments, whereas CKs counteract these effects by promoting cell proliferation and development. This hormonal balance is responsible for the accurate regulation of developmental programs in response to environmental exposures [62].

### 4.4. ABA-JA-SA Interaction

ABA-JA crosstalk is context-dependent and cannot be characterised as uniformly synergistic; its outcome, whether synergistic or antagonistic, is determined by the stress type, tissue, developmental stage, and the relative concentrations and timing of each hormone. Under moderate drought and osmotic stress, ABA and JA exhibit synergistic interactions. Elevated ABA concentrations trigger the degradation of specific JAZ repressor proteins, notably *JAZ3* and *JAZ12*, via a *COI1*-independent mechanism, thereby releasing *MYC2* to activate drought-responsive genes such as *RD22* in root and vegetative tissues [77,78]. Reciprocally, JA application elevates foliar ABA concentrations through AREB/ABF-mediated induction of ABA biosynthetic genes including *NCED3*, reinforcing stomatal closure and osmoprotectant accumulation [79,80]. However, this synergism is not universal. At high ABA concentrations, or under conditions where ABA signalling predominates such as prolonged drought in vegetative tissues, the ABA receptor *PYL6* directly interacts with *MYC2* in an ABA-dependent manner, attenuating *MYC2*-driven JA-responsive gene expression and thereby antagonising JA signalling [81]. Similarly, sustained ABA accumulation can suppress JA-ethylene-mediated defence gene expression, including *PDF1.2*, through *MYC2*-independent repression mechanisms, demonstrating that the two pathways are mutually antagonistic in specific tissues and signalling contexts [82]. The outcome of ABA-JA crosstalk must therefore be interpreted as a dynamic, dose- and context-dependent balance between cooperation and antagonism, rather than a fixed synergistic relationship. In *Arabidopsis*, genetic and biochemical dissection has established that *MYC2* (also designated *JIN1* in *Arabidopsis*) acts as a molecular nexus integrating ABA and JA signals. *AtMYC2* is a direct transcriptional activator of ABA-responsive genes including *RD22* and is additionally required for full JA-induced defence gene expression [83,84]. Loss-of-function *jin1/myc2* mutants display attenuated responses to both ABA and MeJA, confirming the dual regulatory role of *MYC2* in *Arabidopsis*. In crops such as rice and soybeans, orthologous *MYC2* proteins fulfil equivalent functions, linking ABA-JA crosstalk to drought and pathogen tolerance in agronomically relevant species. SA-JA interactions tend to be more complex and context-dependent. While in the presence of biotic stress, SA and JA often act antagonistically, there is evidence that under certain abiotic stresses, SA and JA can show partial synergism, especially when combined with ABA signalling. In drought and salt stress, SA modulates JA-responsive gene expression through NPR1-mediated repression of JA signalling components and competitive interaction at shared promoter elements [16]. Furthermore, ABA and SA interactions balance stress adaptation and growth. ABA frequently inhibits SA accumulation and SA-mediated gene expression under prolonged abiotic stress conditions, thereby promoting water conservation and stress acclimation at the expense of defense responses. This antagonistic interplay ensures efficient resource use during prolonged periods of drought or salinity stress [85].

### 4.5. Brassinosteroids-ABA Crosstalk

BRs are mainly involved in growth and development processes, while ABA mediates adaptive stress responses through multiple convergent signalling pathways. In *Arabidopsis*, the mechanistic convergence of BR and ABA signalling has been most precisely resolved through the *BIN2*-*SnRK2*-*ABI5* axis. Under conditions of BR deficiency or BR signalling inhibition, the GSK3-like kinase *BIN2* is released from repression and directly phosphorylates both *SnRK2.2*/*SnRK2.3* kinases and the transcription factor *ABI5,* creating a convergence node where BR repression potentiates ABA-driven stress gene expression [86]. The BR-responsive transcription factor *BZR1* provides an additional layer of crosstalk by directly interacting with the *ABI5* promoter to repress its transcription, thereby reducing ABA sensitivity in specific developmental contexts such as primary root growth, while *BES1* suppresses *ABI5* transcriptional activity through direct protein-protein interaction, dampening ABA stress signalling under conditions favouring growth [87,88]. Together, these *Arabidopsis* findings establish a model in which BR and ABA pathways share multiple convergence points at the kinase, transcription factor, and protein interaction levels, enabling context-dependent switching between growth and stress adaptation. Conservation and modification of this model have been demonstrated in tomato, where BZR1-mediated transcriptional upregulation of the ABA biosynthetic gene *NCED1* establishes a positive BR-ABA feedback loop that enhances ABA accumulation during chilling stress, indicating that the core *Arabidopsis* regulatory logic is preserved in crop species but with species-specific transcriptional targets [89].

### 4.6. CEP-Hormone Interactions

CEP signalling is integrated with classical phytohormone pathways to control plant adaptation to abiotic stresses, including drought, salinity, and nutrient limitation, as illustrated in Figure 2 [7]. Recent investigations have shown a close interaction between CEP signalling and ABA under abiotic stress conditions. In *Setaria italica*, the *SiCEP3* was shown to enhance ABA signalling by transcriptionally upregulating the expression of genes encoding plasma membrane-localised ABA influx carriers, specifically the ATP-binding cassette transporter *ABCG40* and the nitrate/ABA dual-affinity transporter *NRT1.2*/*NPF4.6*/*AIT1*, as well as the ABA receptor *PYL4*, thereby increasing cellular ABA uptake capacity and amplifying downstream ABA signalling [37]. Combined applications of *SiCEP3* and ABA resulted in greater ABA accumulation and stronger growth inhibition than either treatment alone, demonstrating that SiCEP3 potentiates ABA responsiveness by co-ordinately upregulating both the transport machinery and receptor components of the ABA perception system rather than directly facilitating passive hormone diffusion [37]. In *Arabidopsis*, the interaction between CEPs and auxin signalling has been most precisely characterised through *CEP5*. Loss-of-function *cep5* mutants display enhanced lateral root formation phenocopying high-auxin conditions, while *CEP5* overexpression suppresses lateral root branching, demonstrating that *CEP5* functions as an antagonist of auxin-driven root branching [38]. Proteomic evidence further indicates that *CEP5*-mediated signalling stabilises *AUX/IAA* transcriptional repressors, thereby attenuating *ARF*-dependent transcription and reducing auxin response thresholds in the root, particularly under drought or osmotic stress conditions where *CEP5* activity is associated with increased stress tolerance [38]. Analogous CEP-auxin interactions regulating root architecture under osmotic stress have been documented in wheat and tobacco, suggesting that the *Arabidopsis*-established regulatory module is conserved in agronomically relevant species, though the specific receptor components and downstream targets may differ [7]. In *Arabidopsis*, CEP-ABA crosstalk additionally operates through the *CEPR2-PYR/PYL* phosphorylation module [36]. CEPs additionally interact with cytokinin signalling pathways through the bidirectional *CEP-CEPD* loop (Section 3.1), coordinating root growth and nitrogen uptake in response to environmental cues [39].

### 4.7. Network Complexity

The coexistence of common signalling elements across different hormonal pathways is a fundamental source of network complexity in plant stress signalling. A variety of transcription factors, protein kinases, and secondary messengers are involved in several cascades of hormone signalling, thus allowing intersections and combination of signals that are produced by different signalling pathways. Shared transcription factors including the *WRKY*, *NAC*, and *MYB* families (Section 3.3) serve as central convergence nodes integrating signals from multiple hormonal pathways [90]. Spatiotemporal regulation is another aspect of the complexity of the hormonal networks. The interactions between hormones are often tissue specific and sensitive to the degree of development and the intensity of the stress. Indicatively, the accumulation of ABA in guard cells under drought stress promotes stomatal closure and the interaction of auxin and cytokinins in roots regulates the root system organization to maximize water and nutrient uptake. This kind of spatial control allows plants to produce localized responses without disrupting physiological homeostasis at the whole-plant level [4].

Feedback and feed-forward regulatory loops also increase the complexity of hormonal networks. Most signalling pathways of hormones contain regulatory modules, which stimulate or inhibit signalling through positive and negative feedback mechanisms. As an example, the *PYR/PYL-PP2C-SnRK2* regulatory module (Section 3.1) exemplifies how feedback loops amplify and attenuate ABA signals during stress responses [91]. Furthermore, protein-protein interactions and post-translational modifications (PTMs) provide an extra layer of regulation of hormonal networks. Signalling proteins may be regulated by phosphorylation, ubiquitination and SUMOylation to alter their activity or stability, and thus, affect the interactions between various hormonal pathways. These PTMs allow stress signalling networks to be reprogrammed quickly without the need to alter the expression of genes, which is essential especially when a plant is subjected to a sudden environmental shift [58]. Integrated transcriptomic, proteomic, and metabolomic analyses reveal coordinated multi-hormone regulation of stress responses, enabling a dynamic balance between plant growth and survival [58].

### 4.8. Phytomelatonin-Hormone Crosstalk

Phytomelatonin (PMT) engages in extensive bidirectional crosstalk with classical phytohormones under abiotic stress conditions, positioning it as a central integrative node in the hormonal regulatory network [15,92]. PMT-ABA interactions are among the most extensively characterized. Under drought and osmotic stress, PMT promotes ABA biosynthesis and enhances ABA-mediated stomatal closure through *PMTR1*-dependent activation of ROS and Ca^2+^ signals in guard cells [13]. Reciprocally, ABA upregulates the expression of PMT biosynthetic genes including *SNAT1* and *ASMT1*, creating a positive feedback loop that amplifies antioxidant and stomatal responses during water deficit [93]. Under salinity and drought, PMT also modulates auxin signalling by repressing auxin biosynthesis and polar auxin transport, particularly inhibiting *PIN*-mediated auxin efflux, thereby reducing primary root elongation and promoting lateral root architecture adaptation [94]. PMT-cytokinin interactions are synergistic. PMT delays stress-induced leaf senescence by maintaining cytokinin levels and repressing cytokinin degradation through CKX enzyme inhibition, thereby sustaining photosynthetic capacity under combined osmotic and heat stress [92]. Under high-temperature and drought stress, PMT suppresses ethylene biosynthesis by downregulating ACC synthase (ACS) and ACC oxidase (ACO) gene expression, thereby reducing ethylene-mediated accelerated senescence and promoting stress tolerance [15]. PMT additionally interacts synergistically with JA under combined biotic and abiotic stress. Exogenous PMT upregulates the expression of JA biosynthetic genes including *LOX* and *OPR3*, enhancing JA-mediated antioxidant enzyme expression and secondary metabolite production, while JA in turn stimulates PMT accumulation [15]. The relationship between PMT and SA is context-dependent. Under low concentrations PMT and SA act synergistically to induce systemic acquired resistance and antioxidant defence, while at higher PMT concentrations, SA signalling is partially suppressed to redirect resources toward growth-promoting pathways [92]. Collectively, PMT functions as a master regulatory node that modulates the balance between growth-promoting hormones, including auxins, GAs, and cytokinins, and stress-protective hormones, including ABA, JA, and SA, through both receptor-dependent and receptor-independent mechanisms, enabling fine-tuned whole-plant adaptive responses to diverse abiotic stressors [11,15].

## 5. Integration of Hormonal and Peptide Signalling with Physiological and Biochemical Responses

### 5.1. Stomatal Regulation and Water Use Efficiency

Stomatal aperture and water-use efficiency (WUE) involve hormonal signalling networks, mainly involving abscisic acid (ABA) in times of stress (environmental stress) [91]. ABA-activated SNF1-related protein kinases (SnRK2s) cause anion channel phosphorylation (e.g., *SLAC1*), which causes an anion efflux, membrane depolarisation and subsequent stomatal closure. This signalling cascade reduces transpirational water loss; however, stomatal closure simultaneously restricts CO_2_ diffusion into the leaf, thereby reducing photosynthetic carbon assimilation. Rather than maintaining CO_2_ influx, plants optimise the trade-off between water conservation and carbon gain, a physiological compromise in which the proportional reduction in transpiration typically exceeds the reduction in photosynthesis under moderate stress, resulting in improved intrinsic water-use efficiency [95,96,97]. Other classical phytohormones are involved in stomatal regulation, in addition to ABA, via complex webs of crosstalk. It is important to distinguish between two mechanistically distinct processes. Stomatal aperture regulation, which is a short-term physiological response involving reversible changes in guard cell turgor driven by ion flux, and stomatal development, which is a long-term developmental process involving the asymmetric division and differentiation of stomatal lineage cells. Auxin and cytokinins contribute to both processes but through entirely different mechanisms. With respect to aperture regulation, auxin and cytokinins at physiological concentrations promote stomatal opening and antagonize ABA-induced closure, partly by stimulating ethylene biosynthesis upstream of ACC synthase activity, which in turn suppresses ABA-mediated guard cell shrinkage [98,99]. Ethylene and jasmonic acid do not govern ABA responses to stomatal opening; rather, they modulate ABA-mediated guard cell signalling through mechanistically opposing pathways. Jasmonic acid acts synergistically with ABA to promote stomatal closure. Methyl jasmonate recruits shared ABA signalling components in guard cells including the kinase *OST1/SnRK2.6*, NADPH oxidases *AtrbohD* and *AtrbohF*, and the PP2C phosphatases *ABI1* and *ABI2* and additionally upregulates ABA biosynthesis through induction of *NCED3*, such that MeJA-induced stomatal closure is severely impaired in ABA-deficient mutants [100]. By contrast, ethylene antagonises ABA-induced stomatal closure by promoting the expression and stability of ABI1 and ABI2 PP2C phosphatases, which dephosphorylate and inactivate *SnRK2.6/OST1*, thereby attenuating the ABA-dependent closure cascade [67,98]. The net stomatal aperture thus reflects the integration of these opposing modulatory inputs onto the core ABA signalling machinery, rather than ABA responding to the state of the stomatal pore. By contrast, the role of cytokinin in stomatal development is mechanistically distinct. Cytokinin acts as an endogenous signal that modulates the frequency and type of asymmetric divisions in the stomatal lineage by forming a regulatory circuit with the transcription factor *SPEECHLESS* (*SPCH*), thereby controlling guard cell number and epidermal cell composition rather than aperture dynamics [101]. Empirical evidence has shown that an enhanced plant sensitivity to ABA or improved guard-cell signalling cascade can significantly enhance WUE as well as confer drought tolerance in the economically relevant crop species, including wheat, rice and maize [102]. Recent studies highlight the importance of guard-cell signalling pathways that comprise ROS, calcium ions (Ca^2+^), and nitric oxide (NO) as messengers in the ABA-dependent stomatal closure. These second messengers amplify hormonal signals and regulate downstream ion-channel functions, reinforcing the physiological response to abiotic stress [103].

### 5.2. Photosynthesis and Energy Metabolism

ABA-induced stomatal closure (described in Section 5.1) concomitantly limits CO_2_ availability for photosynthesis. At the same time, ABA signalling upregulates the transcriptional expression of nuclear-encoded antioxidant enzyme genes, including those encoding superoxide dismutase (SOD) and ascorbate peroxidase (APX), through ABRE-mediated gene regulation involving SnRK2-activated transcription factors such as *AREB/ABFs*, thereby enhancing the capacity of the chloroplast to detoxify ROS and limit oxidative damage under stress [45,104]. Photosynthetic electron transport contributes indirectly by generating ROS principally superoxide (O_2_•^−^) at photosystem I and hydrogen peroxide (H_2_O_2_) as byproducts of electron flow. These chloroplast-derived ROS function as retrograde signals that travel from the chloroplast to the nucleus and activate the transcription of stress-responsive antioxidant genes, thereby linking the redox status of the electron transport chain to nuclear gene expression [105]. Oxidative damage and energy homeostasis have been prevented by the coordination of hormonal and biochemical responses. Cytokinins have a complementary influence on chloroplast development and biogenesis, chlorophyll and activity of photosynthetic enzyme in stress conditions [106]. As an example, the exogenous application of cytokinin in rice and wheat promotes the activity of ribulose-1,5-bisphosphate carboxylase/oxygenase (RuBisCO) and increases the photosynthetic efficiency under drought stress [106]. Beyond RuBisCO activity, CKs more broadly activate the transcription and accumulation of photosynthetic proteins, including components of the light-harvesting complexes and the thylakoid electron transport chain, thereby maintaining photosynthetic capacity under mild stress conditions [106]. In contrast, ABA strongly suppresses the transcription of nuclear-encoded photosynthetic genes through ABRE-mediated transcriptional repression, redirecting metabolic resources from photosynthesis toward stress defence during severe water deficit. ABA and jasmonic acid regulate sugar signalling and carbohydrate partitioning by modulating key enzymes of glycolysis and the TCA cycle [93]. In salt stress, compatible solute (proline and soluble sugars) accumulation occurs in response to ABA, which confers osmoprotection and respiratory buffer of energy, maintains ATP production and metabolic plasticity [107]. Jasmonic acid exerts the most potent suppressive effect on plastid gene transcription, downregulating the expression of plastid-encoded photosynthetic genes including those encoding photosystem I and II components through interference with plastid RNA polymerase activity and chloroplast-to-nucleus retrograde signalling, thereby linking defence hormone accumulation to a rapid and substantial reduction in photosynthetic capacity under combined stress conditions [105,108]. Recent integrative studies suggest that photosynthesis and energy metabolism are coordinated in a hormone-dependent retrograde signalling from the chloroplasts and mitochondria to the nucleus. For example, ROS and sugar signals interact with ABA, cytokinin, and jasmonic acid pathways to regulate the expression of nuclear-encoded photosynthetic and metabolic genes, thereby synchronising energy production with stress adaptation [109].

### 5.3. Osmolyte Accumulation

The representative plant osmolytes are proline, glycine betaine, soluble carbohydrates (sucrose and trehalose), and polyols. All these substances are involved in osmotic adjustment and maintenance of cellular integrity in the presence of stressors [110]. Hormonal signalling pathways play a central role in regulating the biosynthesis and accumulation of osmolytes. ABA is a central hormone sensitive to abiotic stress and strongly stimulates the production of osmolytes during drought and salinity. Up-regulated genes associated with signalling of abscisic acid (ABA) include, but are not limited to, the genes that encode enzymes involved in proline biosynthesis including Δ^1^-pyrroline-5-carboxylate synthetase (P5CS) that is the rate-limiting step in the synthesis of proline. The resulting increase in proline accumulation improves osmotic adjustment, macromolecular stabilisation, and redox homeostasis under unfavourable conditions through several mechanistically distinct contributions. Proline does not directly degrade ROS rather, its primary antioxidant-associated roles are indirect. First, as a compatible solute, proline stabilises protein conformation and membrane integrity through kosmotropic effects, thereby reducing the susceptibility of cellular macromolecules to oxidative damage [111]. Second, the proline-P5C metabolic cycle facilitates the transfer of reducing equivalents between cellular compartments, thereby supporting NADP^+^ regeneration and indirectly sustaining the ascorbate-glutathione pathway for H_2_O_2_ detoxification [112]. Third, while proline may contribute modest direct quenching of the hydroxyl radical (•OH) through hydrogen abstraction, evidence for biologically meaningful direct scavenging of O_2_•^−^ or H_2_O_2_ by proline remains limited and debated; its ROS-protective effects are more accurately attributed to the stabilisation of enzymatic antioxidant machinery rather than direct chemical neutralisation of ROS [113,114]. Glycine betaine is a notable osmoprotectant that accumulates in various crop species exposed to salinity and drought stress. It enhances the structural integrity of the proteins and photosynthetic complexes, in particular photosystem II, to protect the photosynthetic apparatus against damage under stress. Empirical data have shown that high levels of glycine betaine stabilize the membranes and increase tolerance to osmotic stress and ionic stress in crops like maize, wheat and barley [115]. Soluble sugars are also useful as osmoprotectants and regulators of metabolism. Sugars, such as sucrose, trehalose, and raffinose, in addition to promoting osmotic adjustment, are signalling molecules that regulate stress-responsive genes. One example is trehalose metabolism that has been associated with improved drought and salinity stress tolerance based on cellular membrane and protein stabilization and maintenance of energetic homeostasis. Polyols such as mannitol and sorbitol aid in osmotic regulation and ROS scavenging by stabilising cellular redox balance and protecting enzymes against denaturation [116,117]. Via hormone-mediated regulation of osmolyte synthesis and metabolic pathways, plants maintain cellular stability, osmotic pressure, and metabolic homeostasis under adverse conditions. This biochemical modification works in harmony with physiological factors, such as stomatal control and photosynthetic acclimation, and thus improves plant survival against abiotic stress.

### 5.4. Antioxidant Defense Systems

Abiotic stresses often lead to excessive accumulation of ROS, including superoxide radical anion (O_2_•^−^), hydrogen peroxide (H_2_O_2_), and hydroxyl radical (•OH). ROS function as signalling molecules under normal cellular conditions in plants, but in excess amounts, these reactive species cause oxidative damage to lipids, proteins, and nucleic acids during stress conditions. Countering this form of damage, plants have evolved complex antioxidant defence systems that are highly regulated by hormone-mediated signalling pathways and are a basic element of abiotic stress tolerance. This antioxidant system includes non-enzymatic and enzymatic components [118]. The main enzyme antioxidants are SOD, catalase (CAT) and ascorbate peroxidase (APX) that sequentially neutralize ROS. The superoxide radical is dismuted by SOD, and the hydrogen peroxide formed is further broken down by CAT and APX into water and oxygen thus limiting oxidative stress in the subcellular structures, including the chloroplasts and mitochondria [45]. The stimulation of antioxidant defenses in response to abiotic stress is hormonally coordinated. ABA was found in studies with wheat cultivars to increase the activity of antioxidant enzymes in drought and salinity. ABA signalling increases the activities of ROS-scavenging enzymes, thereby preserving cellular redox balance and sustaining photosynthetic activity under hostile conditions [119].

Other classical phytohormones such as salicylic acid (SA) and JA are overlapping with ROS signalling pathways. SA can increase the activity of antioxidant enzymes and regulate hydrogen peroxide levels, in contrast to JA-mediated signalling that has been suggested as being involved in the expression of antioxidant defense genes and the improvement in oxidative stress tolerance [120]. Plants also use non-enzymatic molecules such as ascorbate, glutathione, carotenoids, tocopherols, and phytomelatonin to directly neutralise ROS and maintain redox homeostasis. PMT is a particularly potent free radical scavenger, capable of neutralising hydroxyl radicals (•OH), superoxide (O_2_•^−^), and hydrogen peroxide (H_2_O_2_) directly, and its sequential oxidative catabolites cyclic 3-hydroxymelatonin, N1-acetyl-N2-formyl-5-methoxykynuramine (AFMK), and N1-acetyl-5-methoxykynuramine (AMK) retain antioxidant activity, creating a cascade of ROS scavenging reactions that amplify the total antioxidant capacity beyond that of a single molecule [15,121]. Among these non-enzymatic systems, the ascorbate-glutathione cycle is crucial for H_2_O_2_ detoxification in chloroplasts, as well as the maintenance of redox balance in adverse environments [122]. Among these non-enzymatic systems, the ascorbate-glutathione cycle is crucial for H_2_O_2_ detoxification in chloroplasts, as well as the maintenance of redox balance in adverse environments [122].

### 5.5. Growth vs. Stress Trade-Offs

As established in Section 2.1, DELLA stabilisation under stress mechanistically couples’ growth restraint to the activation of stress-protective gene networks in *Arabidopsis*. DELLA proteins interact with JAZ repressors and *EIN3* to redirect transcriptional resources from growth-promoting to defence-promoting programmes, while ABA reinforces this switch by suppressing GA biosynthesis through DELLA-dependent feedback [123]. This regulatory axis is conserved across species, with *SLR1* and *SLRL1* mediating submergence tolerance in rice and GA-DELLA interactions contributing to drought and heat stress tolerance in wheat and maize [124]. Additional layers of growth-defence integration are provided in *Arabidopsis* by brassinosteroid-jasmonate interactions, where BR promotes growth and stress tolerance simultaneously through modulation of photosynthesis and antioxidant status, while jasmonate signalling predominates in defence responses under combined stress conditions, a balance that has similarly been documented in tomato, soybean, and rice [125].

## 6. Classical Phytohormone and Peptide Hormone

**Figure 4:**
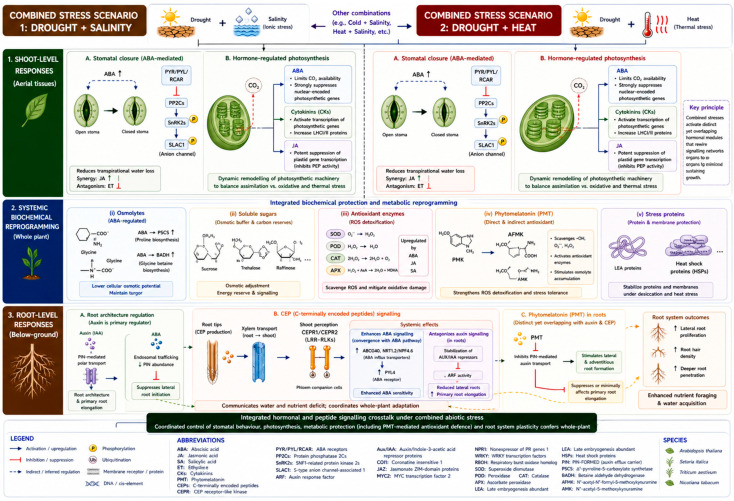
Integrated physiological and biochemical reprogramming of shoot and root systems mediated by classical phytohormone and plant peptide hormone regulatory networks under combined abiotic stress conditions. Plants experiencing combined abiotic stresses such as drought and salinity (**left panel**) and drought and heat (**right panel**) activate a coordinated, whole-plant adaptive response encompassing shoot-level physiological adjustments, systemic biochemical reprogramming, and root-level architectural and foraging remodelling. The dashed vertical axis delineates the two predominant stress combination scenarios, emphasising that combinatorial stresses trigger distinct yet overlapping regulatory modules that collectively rewire signalling networks across organs. Note that other stress combinations, including cold and salinity, activate partially overlapping yet distinct hormonal modules, as described in Section 6. Shoot-Level Responses: In aerial tissues, stress-induced hormonal signals drive two principal physiological adaptations. ABA-mediated stomatal closure (**upper left**) limits transpirational water loss by promoting guard cell contraction through SnRK2-dependent phosphorylation of the *SLAC1* anion channel, a response amplified under drought-salinity combinations where water deficit and ionic toxicity co-occur and further modulated synergistically by jasmonic acid and antagonistically by ethylene. Concurrently, hormone-regulated photosynthesis (**upper right**) involves dynamic remodelling of chloroplast function and photosynthetic machinery: ABA limits CO_2_ availability through stomatal closure and strongly suppresses the transcription of nuclear-encoded photosynthetic genes; cytokinins activate the transcription and accumulation of photosynthetic proteins, including light-harvesting complex components; and jasmonic acid most potently suppresses plastid gene transcription through interference with plastid RNA polymerase activity, collectively reconfiguring the photosynthetic apparatus under drought-heat combinations to balance residual carbon assimilation against oxidative and thermal constraints. Both responses are subject to network rewiring under stress, wherein pre-existing hormonal signalling circuits are reconfigured to prioritise survival while sustaining minimal growth, reflecting transcriptional and post-translational reprogramming of central regulatory hubs. Systemic Biochemical Responses: At the whole-plant biochemical level, stress perception triggers the upregulation of five key protective metabolite and protein classes: (i) osmolytes including proline and glycine betaine, whose biosynthesis is primarily regulated by ABA through transcriptional activation of *P5CS* and betaine aldehyde dehydrogenase genes, lowering cellular osmotic potential to maintain turgor; (ii) soluble sugars including sucrose, trehalose, and raffinose, which serve dual roles as osmotic buffers and carbon reserve signals; (iii) antioxidant enzymes including superoxide dismutase (SOD), peroxidase (POD), catalase (CAT), and ascorbate peroxidase (APX), which neutralise reactive oxygen species (ROS) generated under photoinhibitory and ionic stress conditions, and whose expression is co-ordinately upregulated by ABA, JA, and SA signalling; (iv) phytomelatonin (PMT), which directly neutralises ROS including hydroxyl radicals, superoxide, and hydrogen peroxide through both its parent molecule and its sequential antioxidant catabolites AFMK and AMK, while additionally activating antioxidant enzyme expression and stimulating osmolyte accumulation, functioning as both a direct and indirect antioxidant; and (v) stress proteins including LEA proteins and heat shock proteins, which stabilise protein folding and membrane integrity under denaturing conditions. Root-Level Responses: Below ground, the root system undergoes dramatic structural and functional reprogramming driven by the coordinated activities of multiple hormonal regulators. Auxin, acting through PIN-mediated polar transport, is the primary regulator of root architecture, with ABA modulating PIN protein abundance through endosomal trafficking to suppress lateral root initiation while sustaining primary root elongation for deeper soil penetration. CEPs produced at root tips function as long-distance systemic signals communicating soil water and nutrient deficit to aerial tissues, enhancing ABA signalling through transcriptional upregulation of ABA influx transporter genes and the ABA receptor *PYL4*, and antagonising auxin signalling through *AUX/IAA* repressor stabilisation to further modulate lateral root density. Phytomelatonin (PMT) additionally regulates root architecture by stimulating lateral and adventitious root formation while suppressing or minimally affecting primary root elongation through inhibition of PIN-mediated auxin transport. Together, these hormonal and peptide inputs drive enhanced lateral root proliferation, increased root hair density, and deeper root penetration, enabling spatial exploration of soil moisture and nutrient patches and underpinning enhanced nutrient foraging under resource-limiting combined stress environments. Collectively, this figure illustrates that plant resilience to combined abiotic stresses is not the summation of individual stress responses but rather an emergent property of integrated classical phytohormone and plant peptide hormone regulatory crosstalk that simultaneously coordinates stomatal behaviour, photosynthetic capacity, metabolic protection, including PMT-mediated antioxidant defence, and root system plasticity across the entire plant body. Filled arrows indicate activation or upregulation; blunt-headed lines indicate inhibition or suppression; dashed lines indicate indirect or inferred regulatory connections.

Plants in natural environments are typically not faced with a single type of stress but are often simultaneously exposed to multiple abiotic stresses, such as drought and heat, salinity and high light intensity, or nutrient limitation and water deficit. Crosstalk among classical phytohormones such as ABA, auxin, cytokinins, JA, and ethylene, together with plant peptide hormones (e.g., CEPs), has been identified as a key mechanism that enables the integration of diverse stress signals [126] (Figure 4). Under combined drought and salinity stress, ABA functions as the primary hormonal integrator, coordinating stomatal closure, osmolyte accumulation, and ROS detoxification to limit cellular damage and conserve water. Auxin acts in parallel to modulate root system architecture and growth plasticity, remaining a key regulator of adaptive developmental responses under both water deficit and ionic stress. These two hormonal pathways do not operate independently but are mechanistically integrated. ABA-mediated suppression of *PIN*-dependent auxin transport (Section 4.1), extended under combined stress to include *PIN3* and *PIN7* through *PINOID* kinase-*PP2A* phosphatase balance regulation, limits lateral root initiation while sustaining primary root elongation to enable deeper soil penetration [21,127]. Critically, the primary adaptive function of these root architectural changes is to optimise water and nutrient resource acquisition from deeper, less desiccated soil horizons, not to directly minimise water loss, which is a function of stomatal regulation rather than root development [128,129]. Under combined heat and drought stress, both ABA and JA accumulate in a synergistic manner, collectively coordinating transcriptional and physiological stress responses. In *Triticum aestivum* L., combined heat and drought stress resulted in elevated endogenous levels of ABA and JA, jointly upregulated the activities of antioxidant enzymes (SOD, CAT, and APX; Section 5.4) through transcriptional induction of their corresponding nuclear-encoded genes, and stimulated the accumulation of the compatible solute proline via ABA-dependent *P5CS* gene activation. Collectively, these hormonal and biochemical responses improved intrinsic water-use efficiency, protected photosystem II integrity under heat-induced oxidative load, and enhanced plant survival under combined stress relative to single-stress treatments [130]. Recent studies characterising hormonal and peptide crosstalk in plant responses to major abiotic stress combinations are summarised in Table 1. Cytokinins promote cell division and shoot growth under favourable conditions. However, under combined osmotic stress, cytokinin signalling is actively downregulated rather than simply becoming harmful, a regulated suppression that redirects resources from shoot growth towards stress acclimation. This is not a passive consequence of water loss but a tightly controlled response in which elevated ABA accumulation antagonises cytokinin signalling by phosphorylating and destabilising type-A response regulators, thereby reducing cell proliferative activity in a coordinated manner [71]. Water loss under osmotic stress is therefore not “uncontrolled” but is regulated through stomatal responses, osmolyte accumulation, and root architectural adjustments operating simultaneously. CEPs function as long-distance mobile signals mediating root-to-shoot communication under nutrient limitation and stress. As described in Section 4.6, CEP-ABA interactions under combined drought and salinity are mechanistically nuanced beyond simple induction of ABA signalling. *SiCEP3*-mediated enhancement of ABA signalling in *Setaria italica* (Section 4.6) promotes lateral root branching plasticity under drought while suppressing excessive primary root elongation, enabling optimised water foraging under combined stress [37]. Importantly, CEP-mediated regulation of root architecture under combined stress involves not only ABA but also antagonism with auxin signalling through stabilisation of *AUX/IAA* repressors, as established for *CEP5* in *Arabidopsis*, indicating that the CEP-ABA interaction is embedded within a broader multilayered hormone network rather than constituting a single linear pathway [38]. Emerging multi-omics studies have revealed that plants integrate multiple hormonal cues under combined stresses through shared transcription factors and central regulatory nodes within hormonal signalling networks. The combination of cold and salt stress in *Arabidopsis* induced crosstalk among the three classical phytohormones ABA, ethylene, and JA, mediated by the *ERF* and *NAC* families of transcription factors, to concurrently regulate ROS detoxification, osmolyte accumulation, and stomatal regulation. Similarly, CEPs were shown to regulate both auxin distribution and ABA signalling in the same stress context, thus demonstrating the operation of both classical phytohormones and peptide signals within a highly organised, multilayered network [131].

## 7. Molecular Tools and Omics Approach for Network Analysis

### 7.1. Transcriptomics of Classical Phytohormone and Peptide Hormone-Responsive Genes

RNA sequencing (RNA-seq) and microarray technologies have identified several stress-responsive genes under hormonal control, including ABA, auxin, and jasmonates, as well as plant peptide hormones such as CEPs. These datasets facilitate the identification of candidate transcriptional regulatory networks associated with plant adaptation to environmental stress. However, it is important to note that co-expression patterns and differential gene expression data establish correlation rather than causation. The assignment of regulatory roles to specific transcription factors identified through transcriptomic analysis requires further validation through genetic approaches, including loss-of-function and gain-of-function mutant analysis, chromatin immunoprecipitation sequencing (ChIP-seq), and yeast one-hybrid assays, to confirm direct transcriptional regulation rather than co-regulated responses to a shared upstream signal [154,155]. Transcriptomic analyses in *Arabidopsis* revealed that ABA orchestrates hundreds of drought-responsive genes through the *ABF/AREB*, *NAC*, and *DREB* transcription factors (Section 3.3), regulating osmolyte accumulation, stomatal closure, and ROS detoxification during dehydration [52]. Transcriptomic studies of auxin-treated *Arabidopsis* roots identified an early auxin-responsive gene set comprising the *Aux/IAA*, *GH3*, and *SAUR* families. These genes regulate root architecture by controlling cell elongation, lateral root formation, and auxin transport. Such transcriptional changes are critical for altering root growth patterns under abiotic stress conditions such as drought and nutrient limitation [156]. Transcriptomic analysis of CEP signalling showed that the signalling of CEPs from roots regulates nitrogen transporter gene expression in the shoots and roots. Studies in *Arabidopsis* provided evidence for the perception of CEP activating systemic signalling pathways leading to the induction of nitrate transporter genes such as *NRT2.1*, coordinating nitrogen uptake with the nutritional status of the plant [34]. Transcriptomic analyses of PMT-treated plants under abiotic stress have revealed broad remodelling of hormone signalling gene expression, with upregulation of ABA-responsive, JA-responsive, and antioxidant gene networks, alongside suppression of ethylene signalling genes associated with senescence acceleration [12]. These datasets confirm that PMT functions as a transcriptional integrator of multiple hormonal stress-response pathways rather than as a single-pathway regulator.

### 7.2. Proteomics and Phosphoproteomics

Proteomics and phosphoproteomics give insights into protein abundance and modifications and signalling interactions that are not completely elucidated in transcriptomics. Since several signalling pathways are regulated through post-translational modifications, and in particular phosphorylation, phosphoproteomic analyses have emerged as key tools for the investigation of hormone signalling networks. Phosphoproteomic studies in *Arabidopsis* identified downstream targets of SnRK2 kinases (Section 3.2), including proteins involved in stomatal regulation, transcriptional control, and metabolism. For example, the phosphorylation of the *SLAC1* anion channel controls guard cell ion transport and stomatal closure when plants are under drought stress [157]. Proteomic analysis of tomato plants exposed to salinity stress showed that ethylene signalling modulates the abundance of proteins involved in antioxidant defence and energy metabolism. Enhanced accumulation of proteins involved in ROS detoxification like peroxidases and glutathione-related enzymes demonstrated the role of ethylene in regulating stress-induced proteomes [158]. Phosphoproteomic studies of brassinosteroid signalling networks identified *BZR1* as a key phosphorylation target within receptor kinase cascades, with this phosphorylation regulating gene expression related to growth and stress tolerance [159].

### 7.3. Metabolomics Under Classical Phytohormone and Peptide Hormone Regulation

Metabolomics offers the potential for direct insight into biochemical alterations involved in stress responses in the plant cell. Hormonal and peptide signalling pathways strongly influence metabolic reprogramming, leading to the accumulation of osmolytes, antioxidants, and secondary metabolites that confer greater stress tolerance. Metabolomic studies confirm the ABA-regulated osmolyte accumulation described in Section 5.3, with drought-stressed *Arabidopsis* showing elevated proline, raffinose, and sucrose levels consistent with the transcriptional activation of biosynthetic genes downstream of ABA signalling [160]. JA signalling has been known to control the biosynthesis of defensive secondary metabolites. Metabolomic profiling of JA-treated plants resulted in increased production of flavonoids and phenolic compounds, which are involved in antioxidant defence and protection against oxidative stress [108]. CEP-mediated nitrogen signalling affects metabolic pathways involved in amino-acid biosynthesis and nitrogen assimilation. Metabolomic analysis showed altered levels of amino acids, such as glutamine and glutamate, suggesting systemic metabolic readjustments coordinated by peptide signalling [35]. Metabolomic profiling of PMT-treated plants under drought and salinity stress has revealed elevated levels of proline, soluble sugars, and phenolic antioxidants, alongside reduced accumulation of malondialdehyde as a marker of lipid peroxidation, demonstrating that PMT-mediated metabolic reprogramming integrates osmolyte accumulation and antioxidant enhancement within a coordinated stress-adaptive programme [15].

### 7.4. Systems Biology and Predictive Modelling

Systems biology integrates multi-omics datasets to build a comprehensive picture of the regulatory networks governing plant stress responses. Computational modelling and network analysis are then used to identify key regulatory hubs and predict gene interactions that are involved in hormonal signalling. Systems-level analysis of ABA signalling networks revealed central regulatory modules including *PYR/PYL* receptors, PP2C phosphatases, and SnRK2 kinases. Mathematical modelling showed how feedback loops in this network regulate stress-responsive gene expression and stomatal regulation [91]. Systems biology approaches integrating transcriptomics, proteomics, and metabolomics have revealed candidate interconnected networks associated with plant responses to environmental stress, confirming the central regulatory roles of the *WRKY* and *NAC* transcription factor families described in Section 3.3 [155]. As noted in Section 7.1, network topology inferred from multi-omics data reflects statistical association rather than demonstrated causality; causal validation through loss-of-function mutant phenotyping and ChIP-seq remains essential [154].

## 8. Applications in Crop Improvement

The foundational mechanistic understanding of ABA biosynthesis and signalling, established primarily in *Arabidopsis* through genetic and biochemical dissection of the *NCED-PYR/PYL-PP2C-SnRK2* pathway, has provided the molecular rationale for targeted engineering of drought tolerance in crop species. Transgenic overexpression of *NCED* genes, which encode the rate-limiting ABA biosynthetic enzyme first characterised in *Arabidopsis*, has been translated into improved drought tolerance and water-use efficiency in tomato, tobacco, and maize through facilitation of rapid stomatal closure and enhanced osmotic adjustment [161]. Similarly, the *Arabidopsis*-defined role of *PIN*-mediated polar auxin transport in root architectural plasticity under stress has informed strategies for engineering auxin transport or signalling pathways in crop species to improve root depth, lateral root density, and nutrient acquisition under drought and nutrient-deficient conditions [162]. Root-specific reduction in cytokinin levels through *CKX* overexpression, first demonstrated to enhance root elongation and drought tolerance in *Arabidopsis*, has been successfully applied to generate improved root phenotypes in barley and tobacco, demonstrating the translatability of *Arabidopsis*-derived mechanistic insights to crop improvement [75]. Hormone-centred approaches have been combined with biotechnological tools including genome editing as well as marker assisted breeding. For example, editing genes involved in hormone-related pathways by CRISPR/Cas9 (clustered regularly interspaced short palindromic repeats and associated protein 9) is increasingly being used to create stress-resilient cultivars. Recent investigations have uncovered that fine-tuning of genes involved in hormone signalling cascades can alter stress responses without negatively impacting plant development as a means of providing a sophisticated approach to increase crop tolerance to environmental stressors [163]. Beyond classical phytohormones, CEP hormone pathways have emerged as crop improvement targets, given their dual roles in root architecture and nitrogen acquisition established in Section 2.2 and Section 4.6. Manipulation of CEP pathways is therefore expected to improve nutrient use efficiency and stress tolerance, especially under nutrient limitation or drought [34]. An additional promising strategy is exogenous application of classical phytohormones or their analogues, to enhance the crop performance under stressful conditions. Foliar applications of brassinosteroids, salicylic acid or jasmonates have been found to increase antioxidant activity, maintain photosynthetic efficiency and enhance yield stability under unfavourable environmental conditions. Brassinosteroid application, for instance, can enhance stress tolerance by protecting photosynthetic machinery and stabilising metabolism during the drought and salinity stress [164]. Exogenous application of phytomelatonin represents an additional promising strategy for crop improvement under abiotic stress. Foliar or seed priming applications of PMT have been shown to enhance drought tolerance, salinity resistance, and thermotolerance in agronomically important crops including wheat, maize, rice, and tomato, through combined upregulation of antioxidant enzyme activities, stimulation of osmolyte accumulation, and modulation of ABA and JA signalling pathways [12,15]. The identification of *PMTR1* homologues in multiple crop species provides a molecular target for genetic engineering approaches aimed at enhancing endogenous PMT signalling capacity without relying on exogenous application [12].

Finally, the combination of systems biology and multi-omics has led to the accelerated discovery of hormone regulated genes and regulatory networks involved in stress tolerance. Genomic, transcriptomic, and proteomic analyses can identify candidate regulatory nodes within hormonal signalling pathways that represent potential targets for crop improvement. Omics-derived candidates are subject to the correlation-vs-causation limitation outlined in Section 7.1, translation to breeding targets requires rigorous functional validation in crop species under field-representative conditions, including assessment of pleiotropic effects [74,165]. Where such validation has been performed as in *NCED1* overexpression and CRISPR-mediated hormone pathway editing, the evidence supports causal contributions to stress tolerance. For most candidates, this mechanistic link remains to be established.

## 9. Challenges and Future Directions

One of the main challenges is the inherent complexity and redundancy of hormone crosstalk networks. Multiple classical phytohormones are often involved in regulating overlapping sets of genes and metabolic pathways and include ABA, auxin, cytokinins, jasmonates, salicylic acid, and brassinosteroids. Plant peptide hormones such as CEPs add additional levels of systemic signalling that cross-cut classic hormone signalling pathways. This high level of network redundancy and context dependence of regulation compromises the prediction of phenotypic consequences arising from the manipulation of a single pathway in crop species. For example, the functional dynamics of CEP-ABA or CEP-auxin interactions under field relevant stress combinations is largely unexplored. Addressing this gap in knowledge is critical for engineering crops that have the potential to thrive in complex and multifactorial environmental conditions [74]. A related challenge is the extent to which mechanistic models established in *Arabidopsis* can be reliably extrapolated to crop species. While *Arabidopsis* has provided the foundational molecular framework for hormonal crosstalk, including the *PYR/PYL-PP2C-SnRK2* ABA signalling module, the *TIR1-Aux/IAA* auxin perception system, and the *CEPR1/CEPR2-CEPD* long-distance nitrogen signalling loop, genome duplication, sub-functionalisation of gene family members, and species-specific regulatory elements in crop species such as wheat, rice, and maize frequently modify the precise mechanistic outcomes observed in *Arabidopsis* [74]. Systematic comparative functional genomics studies, combining *Arabidopsis*-established mechanistic models with crop-specific genetic validation, are therefore essential to determine which regulatory nodes are conserved, which are modified, and which are entirely species-specific before translating laboratory findings into breeding programmes. Translating understanding at the molecular and omics level into the field continues to be a major bottleneck. Most studies on hormonal and peptide crosstalk have been done in model species such as *Arabidopsis thaliana* in tightly controlled laboratory or greenhouse conditions. Environmental variability, soil heterogeneity and biotic interactions in natural or agricultural ecosystems can seriously modify the dynamics of hormonal signalling, reducing the efficacy of strategies obtained only from controlled experiments. Additionally, there are the potential trade-offs of genetic manipulation of hormonal pathways. Overexpression of ABA biosynthetic genes or use of the DELLA proteins may have the unwanted consequence of suppressing growth and yield under non-stressful conditions. Fine-tuning the growth-stress trade-off is consequently an important challenge for the practical application of these findings to crop improvement. Future research should focus on several key directions. First, integration of transcriptomics, proteomics, phosphoproteomics, and metabolomics with sophisticated computational modelling can be used to generate hypotheses about the dynamic regulatory architecture of hormone and peptide signalling networks. The correlation-vs-causation limitation described in Section 7.1 applies equally here. The causal validity of network predictions must be tested through genetic perturbation before exploitation for crop improvement [154]. Second, plants in the natural environment are often subject to multiple stresses at the same time; therefore, it is necessary to undertake detailed studies of how classical phytohormone and peptide hormone pathways coordinate responses to multiple stresses. For example, characterising ABA, auxin, and CEP pathway interactions under combined drought-salinity conditions could be a way of finding targets to engineer robust stress resilience [166,167]. Third, field-oriented functional validation taking the discoveries from the laboratory and adapting to breeding programs requires rigorous validation under practical environmental situations. CRISPR/Cas-mediated gene editing, RNA interference, and genome-wide association studies (GWAS) can be used to evaluate candidate genes and regulatory nodes in many different crop species in the field. Many stress responses are both transient and tissue specific. Thus, future innovations in live cell imaging, hormone biosensors, and single-cell transcriptomics will offer fine-scale information about dynamic hormone and peptide signalling under stress. Finally, taking advantage of synthetic biology and peptide analogues is a promising way for crop improvement without compromising crop growth. The design of synthetic peptides or hormone analogues which selectively regulate stress related pathways allows for the fine-tuned modulation of stress responses with minimal undesired trade-offs [168].

## 10. Conclusions

The integration of classical phytohormone pathways with peptide hormone pathways, particularly those mediated by CEPs, constitutes a multilayered regulatory system that enables plants to coordinate adaptive responses across molecular, cellular, and whole-plant levels under abiotic stress. Through these interactions, stomatal regulation, osmolyte synthesis, antioxidative responses, and photosynthetic performance as well as root system structure are coordinated to enhance plant resilience and sustain energetic balance while prioritising survival over unrestricted growth in unfavourable environments. High-throughput omics approaches have identified candidate transcription factors, protein kinases, metabolites, and signalling nodes whose abundance or activity correlates with stress-adaptive classical phytohormone and peptide hormone signalling. While these datasets have substantially advanced the generation of mechanistic hypotheses about the regulatory architecture of stress tolerance, it is important to distinguish between correlative associations identified through profiling studies and causal regulatory relationships that have been validated through rigorous genetic and biochemical experimentation. The fine-scale regulatory processes that determine stress tolerance can only be fully explained where such causal validation has been achieved. For most omics-identified candidates, their precise mechanistic contributions remain to be functionally established. Empirical studies in both model organisms and agronomic crops have shown that it is possible to modulate these pathways to improve crop resilience to stress without any significant impact on growth, which highlights their potential in crop enhancement. The inherent complexity and redundancy of hormonal crosstalk, the fact that little is known about the role of plant peptide hormones in simultaneous stressors, and the persistence of a significant translational gap between laboratory findings and field application represent significant ongoing challenges that must be addressed to fully realise the potential of hormonal and peptide networks for crop improvement. Addressing these challenges will require integrative multi-omics approaches, higher-order computational modelling, and rigorous field-based functional validation, alongside emerging tools such as synthetic biology and precision breeding.

## Figures and Tables

**Table 1:** Recent research studies on hormonal and peptide crosstalk that depict the crosstalk of hormonal and peptide signalling in plant responses to the major abiotic stresses. Such interactions demonstrate regulatory networks that manage stress perception, signalling, and physiological adaptation.

Abiotic Stress	Hormonal/Peptide Crosstalk	Key Regulatory Gene/Signalling Node	Physiological/ Molecular Response	Experimental System	Reference
Drought	GA-ABA	*OsNAC120*	Integration of growth and drought signalling; modulation of ABA biosynthesis genes	Rice	[132]
Drought	BR-ABA	*BIN2* signalling	Enhanced antioxidant activity and improved water use efficiency	Quinoa	[133]
Drought	BR-SA-ABA	Antioxidant pathway genes	Increased osmolyte accumulation and ROS detoxification	Zinnia	[134]
Salinity	Auxin-ROS signalling	*OsARF12*	Regulation of Na^+^/K^+^ homeostasis and ROS scavenging	Rice	[135]
Salinity	Cytokinin signalling	*CKX*-dependent regulation	Stress-dependent modulation of chlorophyll retention and antioxidant activity	Potato	[136]
Osmotic and Salt stress	CEPs	*NtCEP* gene family	Regulation of root growth and osmotic and salt stress adaptation	Tobacco	[137]
Alkaline stress	JA-Auxin	Auxin-related transcriptional regulation	Enhanced root growth and improved alkaline tolerance	Rice	[138]
Flooding	Ethylene-ABA	Ethylene-responsive genes	Adaptive transcriptomic changes in roots under flooding	*Styrax japonicus*	[139]
Cold	BR-ABA	*NCED1* regulation	Enhanced ABA biosynthesis and improved cold tolerance	Tomato	[89]
Cold	ABA-ERF15	*ERF15-CBF-WRKY* module	Activation of cold-responsive genes and increased freezing tolerance	Tomato	[140]
Cold	ABA-JA-SA	Hormone signalling network	Organ-specific hormonal responses in leaves and roots	Rice	[141]
Heat stress	ABA signalling	*OsPRMT6b*	Feedback regulation of ABA signalling during heat recovery	Rice	[142]
Multi-stress	JA signalling	*JAUP1* gene	Root development and stress tolerance via jasmonate signalling	Rice	[143]
Nutrient stress/Nitrogen signalling	CEP-cytokinin	*CEPD* glutaredoxins	Regulation of root growth under nutrient limitation	*Arabidopsis*	[39]
Nutrient/root growth regulation	CEP-auxin-cytokinin	CEP signalling pathway	Root system architecture remodeling	*Arabidopsis*	[144]
Abiotic stress/nutrient-linked adaptation	CEP-auxin-sugar	*OsCEP8*	Integration of hormone and sugar signalling during stress	Rice/*Arabidopsis*-based system	[145]
Nitrate fluctuation	Cytokinin biosynthesis	*IPT3* epigenetic regulation	Adjustment of cytokinin production and root growth	*Arabidopsis*	[146]
Nitrate + light cue integration	Cytokinin-light signalling	Phytochrome-dependent pathway	Shoot elongation coordination with root cytokinin signals	*Arabidopsis*	[147]
Phosphate deficiency	Iron-root signalling	*CYBDOM* protein	Regulation of root growth and Fe homeostasis	*Arabidopsis*	[148]
High light stress	SA-JA	ROS wave signalling	Systemic acquired acclimation through ROS signalling	*Arabidopsis*	[149]
Cadmium stress	SA signalling	SA-dependent defense genes	Reduced Cd accumulation and improved antioxidant defense	Spinach	[150]
Cadmium stress	JA-SA	Hormone interaction network	Increased selenium uptake and reduced Cd toxicity	Pak choi (*Brassica chinensis* L.)	[151]
Nickel stress	SA-JA	Metal-responsive genes	Enhanced phytoremediation efficiency	*Alyssum inflatum*	[152]
Combined drought + Cd	SA signalling	Stress defense network	Improved tolerance to simultaneous drought and metal stress	*Pterocarya fraxinifolia*	[153]

## Data Availability

No new data were created or analysed in this study. Data sharing is not applicable to this article.
